# Aerosol-Assisted Crystallization Lowers Intrinsic Quantum Confinement and Improves Optoelectronic Performance in FAPbI_3_ Films

**DOI:** 10.1021/acs.jpclett.5c00041

**Published:** 2025-02-21

**Authors:** Gurpreet Kaur, Madsar Hameed, Jae Eun Lee, Karim A. Elmestekawy, Michael B. Johnston, Joe Briscoe, Laura M. Herz

**Affiliations:** † Clarendon Laboratory, Department of Physics, University of Oxford, Oxford OX1 3PU, United Kingdom; ‡ School of Engineering and Materials Science, Queen Mary University of London, Mile End Road, London E1 4NS, United Kingdom; § Department of Chemical & Polymer Engineering, University of Engineering & Technology Lahore, Faisalabad Campus, 3.5km, Khurrianwala - Makkuana By-Pass, Faisalabad 39161, Pakistan; ∥ Institute for Advanced Study, Technical University of Munich, D-85748 Garching, Germany

## Abstract

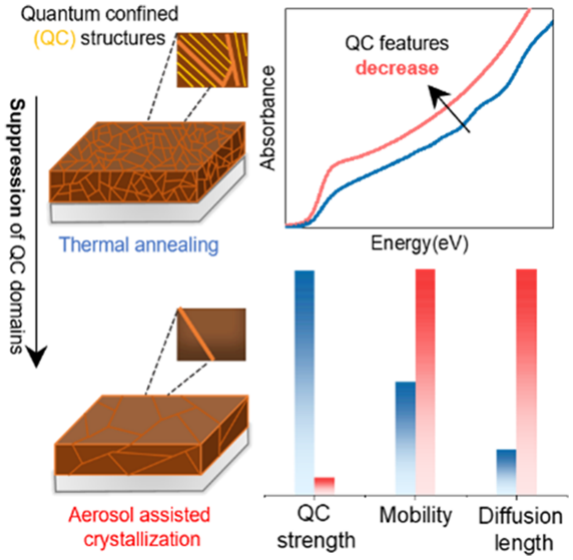

FAPbI_3_ has emerged as a promising semiconductor for photovoltaic applications offering a suitable bandgap for single-junction cells and high chemical stability. However, device efficiency is negatively affected by intrinsic quantum confinement (QC) effects that manifest as additional peaks in the absorption spectra. Here, we show that aerosol-assisted crystallization is an effective method to improve crystallinity and suppresses regions exhibiting QC in FAPbI_3_. We demonstrate that films with minimized QC effects exhibit markedly enhanced optoelectronic properties, such as higher charge-carrier mobilities and recombination lifetimes. Films crystallized under an aerosol solvent flow of either a mixture of *N*,*N*-dimethylformamide and dimethyl sulfoxide or methylammonium thiocyanate vapor displayed reduced charge-carrier recombination losses and improved diffusion lengths compared to those of thermally annealed control films. Our study indicates clear correlations between suppression of QC features in absorption spectra with optimization of crystallinity and mitigation of internal strain, highlighting pathways toward high-performance solar cells.

Metal halide perovskites (MHPs) have dramatically redefined the landscape of photovoltaic (PV) devices, with power conversion efficiencies (PCEs) surging from 3.8% in 2009 to >26% by 2024, showcasing their extraordinary potential in a remarkably short time frame.^1−3^ This impressive leap in efficiency is propelled by the distinctive properties of MHPs, including their finely tunable bandgaps, prolonged hot-carrier cooling times, high absorption coefficients, defect tolerance, excellent charge transport, and ease of fabrication.^4−15^ Within the MHP family, FAPbI_3_ (formamidinium lead iodide) is particularly noteworthy for its optimal bandgap near 1.48 eV, aligning closely with the ideal value for single-junction solar cells, thereby enabling optimized solar absorption.^16−18^ Furthermore, FAPbI_3_ exhibits superior chemical stability of FA compared to those of other hybrid counterparts, which is a crucial factor in addressing the stability challenges that have historically affected perovskite solar cells.^19−21^ These attributes collectively render FAPbI_3_ a prime candidate for developing sustainable, high-efficiency PV technologies and securing a pivotal role in future renewable energy solutions.

Notwithstanding, a major obstacle to commercial adoption remains. FAPbI_3_ still falls short in achieving the long-term structural stability required for practical, real-world applications. The desired α-phase is metastable at room temperature and prone to transition to the non-perovskite δ-phase under prolonged exposure to operational conditions, a problem exacerbated by moisture and thermal stress.^19−22^ While a multitude of strategies such as cation^23−26^ and halide mixing,^27−29^ additive incorporation,^30−33^ and encapsulation^34−36^ have been employed to address these challenges, they often introduce new complexities, including altered bandgaps, halide segregation, and defects, limiting their effectiveness.^37−39^ One promising recent advance in this area has been aerosol-assisted crystallization (AAC), which, as an entirely additive free approach, has the potential to induce stability without such drawbacks. AAC works by directing a laminar flow of nitrogen carrying a fine mist of Lewis base aerosol mixtures over the surface of a freshly cast perovskite film heated to a specific temperature. As the aerosol vaporizes, it penetrates the perovskite layer, encouraging intergrain mass transport and facilitating grain growth via the Ostwald ripening process (i.e., larger grains grow at the expense of smaller ones).^40−42^ This controlled crystallization mechanism induces grain growth in a more uniform manner, ultimately resulting in films with an enhanced structural consistency and higher quality. The real strength of AAC lies in its ability to exploit the interaction between Lewis base solvents and Pb^2+^ ions, which induces octahedral tilting in the perovskite structure.^40,43^ This tilting is crucial as it drives transitions from the unwanted face-shared δ-phase to the desirable, corner-shared cubic α-phase of FAPbI_3_. Thus, AAC facilitates this required phase transition at lower temperatures compared to conventional thermal annealing, effectively reducing in-plane tensile strain and resulting in enhanced crystallinity and more phase-stable films.^41^ Briscoe et al. recently investigated photovoltaic devices based on FAPbI_3_ absorber films produced via the AAC route (DMF/DMSO solvent aerosol) and demonstrated notable improvements in both performance and stability in contrast to thermally annealed (TA) films. Photovoltaic devices showed a synergistic enhancement in photovoltaic parameters with AAC, with average champion PCE values increasing from 15.6% (TA route) to 20.2% when changing from the TA route to the AAC route. Additionally, long-term stability measurements revealed that while the performance of PV devices based on FAPbI_3_ films produced via the TA route degrades sharply within the initial 100 h, AAC-based devices exhibit significantly delayed aging, confirming their superior durability.^44^ Similarly, Lu et al. reported that MASCN vapor treatment results in devices with remarkable PCE values exceeding 23%.^45^ In addition to the notable efficiencies, both studies also reveal significantly enhanced stability in the prepared films, addressing one of the pressing challenges in the commercial viability of FAPbI_3_ solar cells.^44,45^

Beyond the challenges mentioned above in terms of structural stabilities, and potentially linked to them, FAPbI_3_ also demonstrates a distinctive and unusual characteristic not observed in other MHPs, a natural tendency to exhibit intrinsic quantum confinement (QC) effects, revealed by the presence of distinct peaks or undulations in the absorption spectrum above the absorption onset.^46,47^ The basis of these effects has been hypothesized to stem from the formation of nanostructured domains functioning akin to quantum wells or superlattices within the ostensibly bulk film.^46,47^ The prevailing theory posits that these features originate from thin layers of the semiconducting α-FAPbI_3_ phase self-organizing into superlattices or quantum wells with adjacent regions functioning as energy barriers. This self-organization could function as a stress-relief mechanism, with these energy barriers possibly arising from thin layers of the δ-phase or other secondary phases.^46−48^ We note that the band alignment between such intrinsic QC domains and the surrounding bulk FAPbI_3_ phase is most likely of a type I nature. Previously reported low-temperature photoluminescence spectra of FAPbI_3_ exhibit weak sharp features arising from intrinsic QC, while time-resolved PL measurements indicate funneling of both types of charge carriers from the QC domains to the surrounding bulk FAPbI_3_ phase.^46^ While such QC effects may benefit applications such as light-emitting diodes (LEDs), which require strong electron–hole wave function overlap, they pose significant challenges in PV devices, for which efficient charge separation and unimpeded percolation pathways are essential. The formation of QC domains, even in small pockets within the absorber layers, can greatly hinder the smooth flow of photocurrent, resulting in a noticeable decrease in the overall efficiency of solar cells.^46,49^ Therefore, for the effective deployment of FAPbI_3_ in PV devices, it is crucial to strategically mitigate or eliminate these confined regions to preserve and enhance device performance. In this context, it is particularly interesting to understand whether the recent PCE gains for solar cells based on FAPbI_3_ produced via the AAC route may be associated with morphology refinement and whether concomitant alterations in QC domains may play a role. Elmestekawy et al. previously explored how variations in crystallization route influence the formation of QC domains, showing a direct impact on PCE values.^49^ However, no study has unraveled how AAC affects QC domain formation in FAPbI_3_ or whether observed efficiency gains result from a synergistic effect of morphology refinement combined with favorable changes in QC effects.

To address this existing knowledge gap, our study examines how the AAC approach affects the presence of intrinsic quantum confinement effects in FAPbI_3_ and its optoelectronic parameters, which ultimately guide device performance. For this purpose, we fabricated FAPbI_3_ films through three types of fabrication protocols: using conventional thermal annealing as the control (FAPbI_3_ control) and using AAC with two different Lewis base solvent mixtures, DMF/DMSO (DMF, *N*,*N*-dimethylformamide; DMSO, dimethyl sulfoxide) and MASCN (methylammonium thiocyanate) in DMF, termed FAPbI_3_ DMF/DMSO and FAPbI_3_ MASCN, respectively. These solvent aerosols were deliberately chosen for their varying basicity, as this affects their binding affinity with Pb^2+^ ions and thus the crystallization dynamics,^50^ which may lead to differences in QC domain formation. Through analysis of the high-energy region in the absorption spectra, we find a striking contrast between the prominence of QC effects between the control and AAC-prepared films. While the control film exhibits strong QC effects, evident from prominent high-energy peaks, these features are shown to be either significantly reduced (FAPbI_3_ DMF/DMSO) or entirely absent (FAPbI_3_ MASCN) for AAC-prepared films. The AAC approach thus substantially mitigates QC domain formation, with the choice of the solvent aerosol mixture playing a critical role in determining the extent of this suppression. Using time-resolved photoluminescence and terahertz photoconductivity techniques, we further show that such suppression of QC effects corresponds with clearly improved optoelectronic performance that correlates with enhanced crystallinity and reduced strain. AAC-prepared FAPbI_3_ films exhibit reduced charge-carrier recombination rates, higher mobility, and extended diffusion lengths. Collectively, these findings highlight the power of the AAC approach for the suppression of QC and enhancement of optoelectronic quality in FAPbI_3_ films and reveal links between QC domain formation and material crystallinity and strain.

The three stoichiometric bulk FAPbI_3_ films investigated in this study share consistent initial steps in terms of precursor solution preparation and deposition onto the substrate but are then processed differently as outlined above (full details are provided in the Supporting Information). We begin by investigating how different crystallization approaches impact the overall morphology. Scanning electron microscopy (SEM) images in Figure 1a–c clearly illustrate pronounced differences in the average lateral grain sizes across the set of three films. While the thermally annealed (TA) FAPbI_3_ control film exhibits a fine grain structure with small, closely packed grains, averaging 1.0 ± 0.2 μm in size, the FAPbI_3_ films grown through aerosol assistance with DMF/DMSO exhibit moderately larger grains, averaging 1.5 ± 0.4 μm, and with MASCN significantly larger grains with an average size of 4.6 ± 1.7 μm. It is important to note that these grain size estimates derived from SEM analysis (shown in Figure S1) should be considered as upper limits because SEM does not reveal any internal lattice defects, deformation, or strain.^51^ The observed variations can be attributed to the differing basicity of the Lewis base solvents used in the AAC route, namely, DMF/DMSO and MASCN, which lead to distinct crystallization growth rates.^52^ To verify if the fabricated films crystallized in the desired α-phase, X-ray diffraction (XRD) analysis was conducted, which also revealed further information about crystallinity through peak width analysis. Figure 1d shows that the diffraction angles (2θ) of the prominent peaks observed in the data sets for all three films align well with the standard reference peak pattern for the pseudocubic perovskite structure of FAPbI_3_, as indicated by the simulated pattern from the provided reference^61^ (see the Supporting Information for details) and suggesting accurate stoichiometry. However, the full width at half-maximum (fwhm) of the diffraction peaks (Figure S3) decreases in the following order: control, DMF/DMSO-treated, and MASCN-treated FAPbI_3_ films. The reduction in peak widths offers valuable insight into crystallite size, with narrower peaks indicating larger crystallites according to the Scherrer equation (eq S1).^53^ Our Scherrer analysis yields crystallite sizes of 40 nm for the TA FAPbI_3_ control and larger sizes for the aerosol-assisted growth of FAPbI_3_ with DMF/DMSO (80 nm) and MASCN (82 nm). We note that these reported values represent a lower limit of the actual grain size, as XRD quantifies crystallite size, which is typically smaller, and individual grains can comprise multiple crystallites. Combining observations from both SEM and XRD, however, clearly reveals enhanced crystallinity in the FAPbI_3_ films prepared via the AAC method, with MASCN being even more effective as an aerosol than DMF/DMSO.

**Figure 1:**
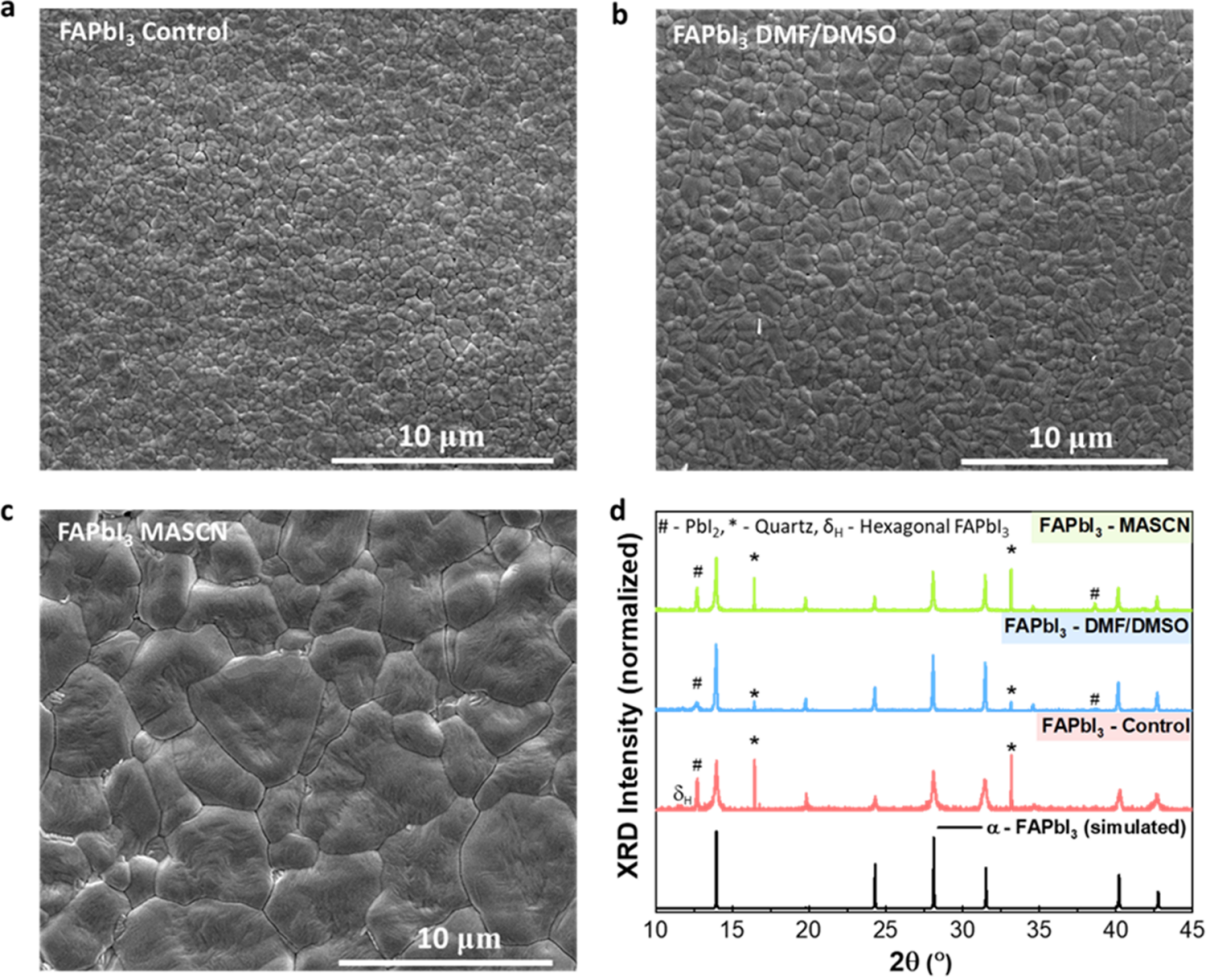
Structural and morphological characterization of FAPbI_3_ films crystallized through different approaches, a thermally annealed control and an aerosol-assisted crystallization method based on either DMF/DMSO or MASCN in DMF. (a–c) Top-down SEM images of the as-prepared thin films on quartz substrates. (d) XRD patterns of the investigated films (measured using a Cu Kα source) contrasted against the reference pattern simulated for the pseudo cubic (α) phase (see the Supporting Information for details). Peaks labeled as *, #, and δ_H_ correspond to the substrate (quartz), lead iodide (PbI_2_), and non-perovskite/hexagonal phase of FAPbI_3_, respectively.

We proceeded by investigating how such morphological differences between FAPbI_3_ films induced by the AAC method influence the prominence of intrinsic QC. To quantify the presence of QC, we followed the previously published methodology of analyzing the peaks observed in the absorption coefficient spectrum above the absorption onset, which have been previously identified as the signature of the QC phenomenon in bulk FAPbI_3_ films.^46,47,49^ We derived absorption coefficient spectra for each film (Figure 2a) from transmission (T) and reflectance (R) spectra recorded with a Fourier transform infrared spectrometer (FTIR) (data processing details are given in the Supporting Information). To proceed with our analysis, we first determined the optical bandgap (*E*_g_) for all three FAPbI_3_ films by applying Elliott fits^54,55^ to the absorption edges (Figure S4). Further details are provided in the Supporting Information, with the fitting parameters listed in Table S1. Bandgaps derived from the fits exhibit a small but discernible progressively decreasing trend from the FAPbI_3_ control (1.563 eV) to the DMF/DMSO (1.552 eV) and MASCN FAPbI_3_ (1.547 eV) films. This decrease in bandgap corelated with our findings of increasing crystallinity along this series and likely results from decreasing in-plane tensile strain, in agreement with similar findings of Du et al.^44^

**Figure 2:**
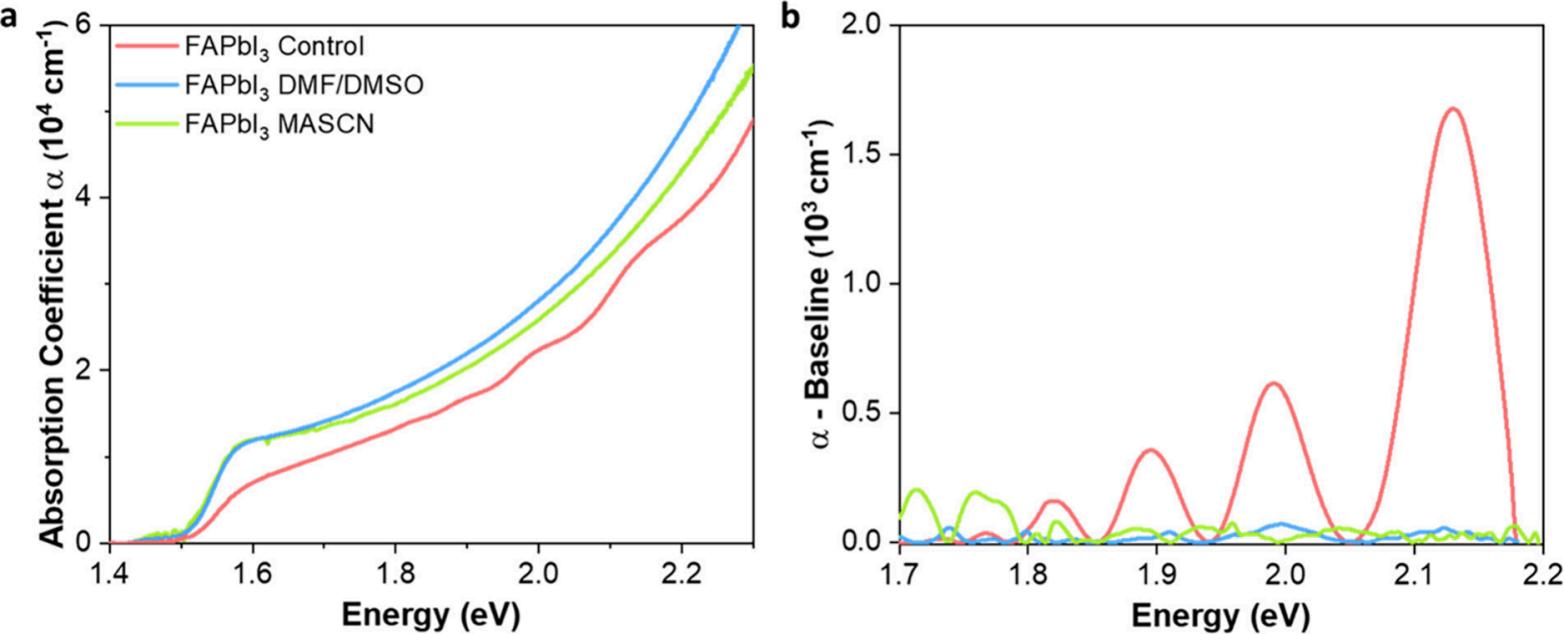
(a) Absorption coefficient spectra and (b) extracted peak features above the absorption onset for FAPbI_3_ films prepared by three methods. The legend of panel a also applies to panel b.

To examine the presence of QC, we focus on the spectral profiles in the region above the bandgap. Figure 2a reveals that the FAPbI_3_ control film displays distinctly identifiable peak features characteristic of QC, which are superimposed on the increasing absorption spectrum, while these features are far less pronounced in the films prepared through the AAC route. To quantify the extent to which QC is present, we implemented a phenomenological fitting method to isolate the peak features otherwise superimposed on the absorption coefficient spectrum, following the methodology previously reported (discussed in Figure S5).^46,47^ For the FAPbI_3_ control film, the resulting decoupled peaks (Figure 2b) display the previously observed increases in amplitude and peak spacing with an increase in energy. Because QC is prominent in this film, we use the decoupled peaks to determine a so-called confinement energy (*E*_Peak_ – *E*_g_), which comprises the difference between the extracted peak energies (*E*_Peak_) and the bandgap energy (*E*_g_) determined from Elliott fits. The confinement energy is found to follow a quadratic relationship with the peak indices (*m*) (Figure S6), in agreement with previous reports,^47^ as would be expected in the presence of electronic confinement in a two-dimensional quantum well or superlattice.^56,57^ For the FAPbI_3_ films processed via the AAC route, these effects are clearly much weaker. To establish whether for these films the decoupled peak data shown in Figure 2b are also the outcome of intrinsic QC effects that are simply obscured by noise and their low amplitude (see Figure S7) or are instead merely random fluctuations, we performed a correlation analysis (see the Supporting Information for details and Figures S8 and S9) between peak positions observed across three FAPbI_3_ films. The outcome indicates that for the FAPbI_3_ film processed with DMF/DMSO, very weak features associated with QC effects persist, while for the film processed with MASCN, no signature related to the presence of QC could be established.

The results compellingly reveal how crystallization dynamics and fabrication methods impact intrinsic quantum confinement effects in nominal bulk FAPbI_3_ films. Correlating our examination of absorption spectra with our analysis of SEM and XRD data, we find that the variation in QC features can be traced back to the different levels of crystallinity and residual tensile strain introduced during fabrication. The control film, produced via thermal annealing, retains significant strain,^44^ which explains the pronounced QC features seen in its optical spectra. In contrast, films prepared using the AAC route, designed to relieve strain, showed a notable reduction in QC effects. Interestingly, the DMF/DMSO-based film still displays faint QC features, likely because it retains more strain than the MASCN-based film, which exhibits minimal strain and no discernible QC effects. These findings paint a clear picture of the strong link between reduced strain and weakening of QC features, illustrating how strain may play a crucial role in the formation of QC structures.

Considering these innate variances in the morphological and electronic landscapes of the FAPbI_3_ films, it is reasonable to expect that the ensuing charge-carrier transport and overall dynamics may differ significantly across these films. Defect densities may also be directly affected by fabrication routes, which increase the extent of nonradiative recombination and negatively impact PV performance. As a preliminary assessment, we examine steady-state photoluminescence (SSPL) spectra shown in Figure 3a for the three types of FAPbI_3_ films, recorded under identical excitation conditions for all samples in a low-pressure environment (∼10^–2^ mbar) at room temperature with an excitation wavelength of 398 nm. These spectra show that FAPbI_3_ films prepared through the AAC method exhibit significantly stronger emission than films fabricated using the conventional TA approach, with the MASCN crystallization-assisted FAPbI_3_ film emitting the most efficient PL. This outcome likely results from a twofold benefit conferred by aerosol treatment. (1) As revealed in the preceding sections, the AAC route produces films with larger grains and fewer grain boundaries, reducing the likelihood of charge carriers scattering or becoming trapped at nonradiative recombination sites that tend to be particularly prominent at grain surfaces.^44,58^ (2) The efficient surface passivation provided by the Lewis bases in the aerosol solvent mixtures further decreases the surface defect density minimizing nonradiative recombination pathways.^67,68^ These effects also have a beneficial impact on the energetic disorder encountered by charge carriers, as observed through a narrowing of PL line widths, i.e., 0.12 eV for the FAPbI_3_ control and 0.1 eV for DMF/DMSO- and 0.09 eV for MASCN crystallization-assisted FAPbI_3_.

**Figure 3:**
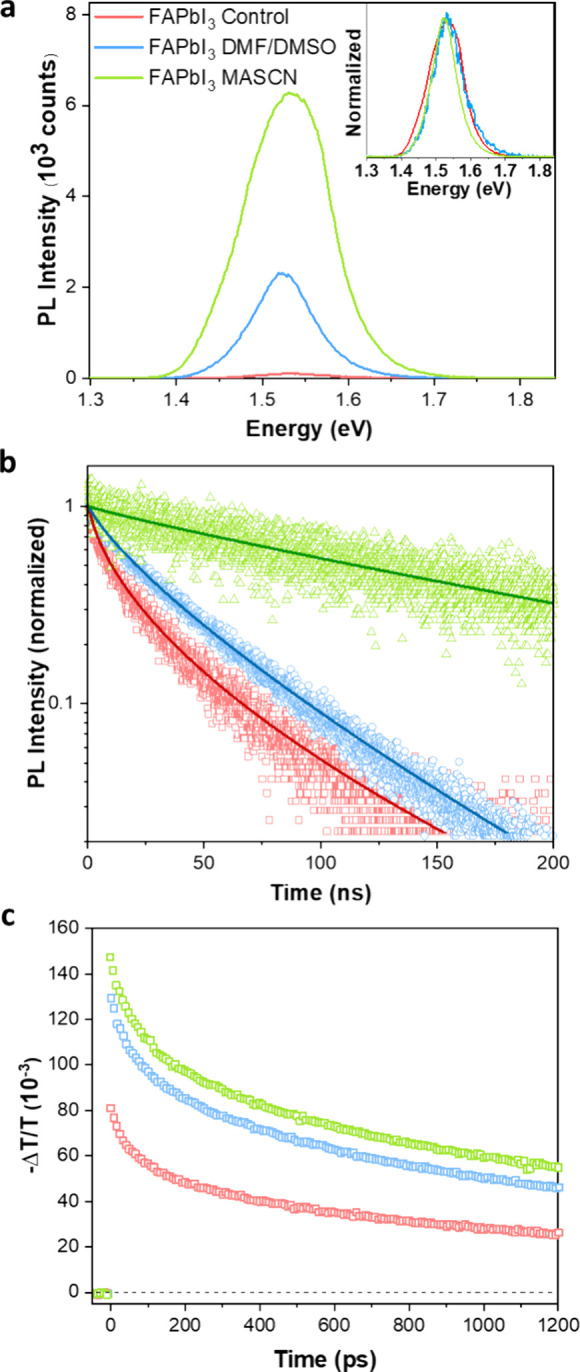
Photoluminescence spectra, corresponding transients, and optical-pump terahertz-probe (OPTP) transients for FAPbI_3_ films produced through three methods. (a) Steady-state PL spectra following excitation with a continuous-wave laser source operating at 398 nm (excitation intensity of 70 mW/cm^2^). The inset displays normalized spectra to visualize the changes in PL peak energy across different films. (b) TRPL transients recorded at the PL peak maxima for each film following photoexcitation by a 398 nm pulsed diode laser source (repetition rate of 1 MHz) with an excitation fluence of 14 nJ/cm^2^. Dynamics for other fluences are available in the Supporting Information. Experimental data are represented by empty symbols, while solid lines denote the corresponding stretched exponential fits from which monomolecular recombination rates (*k*_1_) are extracted. (c) OPTP transients obtained after photoexciting the films with a 3.1 eV optical pump (the excitation fluence for the data set shown here is 28 μJ/cm^2^, and data acquired at other fluences are provided in the Supporting Information).

To further quantify the presence of various charge-carrier recombination pathways, we conducted time-resolved photoluminescence and photoconductivity studies for a range of excitation fluences. In our analysis, we include three primary mechanisms contributing to charge-carrier recombination: (i) Shockley–Read–Hall (SRH) recombination, characterized by rate constant *k*_1_, (ii) bimolecular electron–hole band-to-band recombination, described by *k*_2_, and (iii) Auger recombination, characterized by rate constant *k*_3_. As a first approximation, the resulting charge-carrier dynamics may be described by the following rate equation:^59,60^
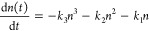
1

We note that because each of the three processes depends on charge-carrier density *n* to a differing extent, analysis of excitation density-dependent transients can unravel the separate processes.

To focus first on the analysis of the trap-mediated SRH recombination channel, we performed TRPL in the low-fluence regime for which these processes dominate. Figure 3b shows the PL transient decay curves recorded at the lowest fluence employed (14 nJ/cm^2^) for all three FAPbI_3_ films (measurements across a range of fluences are provided in Figure S10), enabling a comparison across the three film fabrication protocols. To extract decay times, we fitted these data using a stretched exponential model,^61^ described by the decay function *I* = *I*_0_ exp[−(*t*/τ_C_)^β^], represented by the solid lines. This model accurately captures the deviation from monoexponential decays resulting from the presence of a broad distribution of recombination lifetimes associated with non-uniform trap site distributions and other inhomogeneities. Here, τ_C_ represents the characteristic lifetime, and β, ranging between 0 and 1, indicates heterogeneity,^62^ with values near 1 indicative of a lack of heterogeneity. Recombination rate *k*_1_ is then derived from the relation , where Γ is the gamma function.^63^ More detailed discussion on the fitting procedure is provided in sections 10 and 12 of the Supporting Information. The extracted values for *k*_1_ (listed in Table 1) show that FAPbI_3_ films fabricated through MASCN-assisted crystallization exhibit by far the slowest recombination dynamics, followed by those of DMF/DMSO-assisted FAPbI_3_, with the FAPbI_3_ control film displaying the shortest charge-carrier lifetime. These trends are consistent with trap-mediated nonradiative SRH recombination being largely proportional to the density of defect states, making it highly sensitive to film quality.^64−66^ Our findings highlight that MASCN-assisted crystallization is highly effective in suppressing SRH recombination, in agreement with the observed larger grain sizes and lower strain, which also positively contribute to the absence of QC effects. This conclusion is further supported by the β values listed in Table S2 that reveal that MASCN-assisted FAPbI_3_ exhibits the highest β and is therefore the most ordered with the fewest inhomogeneities, in contrast to the highly heterogeneous charge-carrier decay associated with the FAPbI_3_ control film.

**Table 1:** Extracted Values of Grain Sizes, Spectral Areas Associated with the QC Absorption Peaks (QC S. area), Recombination Constants (*k*_1_–*k*_3_), Effective Electron–Hole Sum Mobilities (φμ), and Diffusion Lengths (*L*_D_) for FAPbI_3_ Films Processed via Thermal Annealing (control) or either DMF/DMSO- or MASCN Aerosol-Assisted Crystallization^a^

	FAPbI_3_ control	FAPbI_3_ DMF/DMSO	FAPbI_3_ MASCN
crystallite size (nm) (XRD)	40	80	82
grain size (μm) (SEM)	1	1.5	4.6
QC S. area (%)	1.4	0.06	0.16
*k*_1_ (s^–1^)	45.6 × 10^6^	27.4 × 10^6^	5.5 × 10^6^
φ*k*_2_ (cm^3^ s^–1^)	1.2 × 10^–10^	0.25 × 10^–10^	0.24 × 10^–10^
φ^2^*k*_3_ (cm^6^ s^–1^)	4.00 × 10^–30^	0.62 × 10^–30^	0.96 × 10^–30^
φμ (cm^2^ V^–1^ s^–1^)	25.1 ± 1.6	39.1 ± 1.3	49.1 ± 2.0
*L*_D_ (μm)	1.16	1.89	4.65

a *k*_1_ values derived from stretched exponential fits to TRPL transients recorded for the lowest fluence employed (14 nJ/cm^2^). Values alternately derived under consideration of higher-order recombination do not vary significantly (see the Supporting Information). *L*_D_ values presented are for the charge-carrier density of 10^16^ cm^–3^ approximately equivalent to 1 sun illumination (assuming φ = 1).

We proceed by examining the higher-order recombination channels occurring in these FAPbI_3_ films, i.e., bimolecular recombination, which typically contributes at moderate carrier concentrations (10^16^–10^18^ cm^–3^), and Auger recombination, which becomes significant at significantly increased densities (>10^18^ cm^–3^).^10,67^ For this purpose, we recorded fluence-dependent photoconductivity transients using the OPTP technique, varying the initially photoexcited charge-carrier densities between 1 × 10^16^ cm^–3^ and slightly greater than 1 × 10^18^ cm^–3^. We note that bimolecular band-to-band recombination is an inverse absorption process^68^ and hence reliant on the electronic band structure encountered by charge carriers (while also being influenced by photon re-absorption events^69^). Similarly, Auger recombination depends on the band structure as a result of the requirement for energy and momentum conservation during the three-particle process.^69−71^ As a consequence of this, one might expect a clear relationship between bimolecular and Auger recombination rate constants and the presence of intrinsic QC domains, which exhibit a modified electronic structure, which is evident from the superimposed peaks in the absorption spectra. Figure 3c presents the time-dependent change in the transmitted terahertz electric field amplitude (Δ*T*/*T*) for three FAPbI_3_ films collected under identical excitation conditions [pump excitation energy of 3.1 eV, fluence of 28 μJ/cm^2^ (*n* ≈ 10^18^ cm^–3^)]. Here, Δ*T*/*T* is proportional to the induced photoconductivity and therefore to charge-carrier density *n*. We therefore used sets of fluence-dependent transients recorded for each type of FAPbI_3_ film (shown in Figure S11) to extract values for *k*_2_ and *k*_3_ from global fits based on the rate equation presented above (eq 1). For these fits, we set the value of *k*_1_ to that determined earlier from TRPL transients collected over longer time scales and recorded at low fluence (see Table 1) for which higher-order recombination processes are less significant. Considering the 1.2 ns measurement window for the photoconductivity transients, the monomolecular recombination lifetimes are too long to meaningfully affect the fitting results (see the Supporting Information for a comprehensive discussion of the fitting details). We note that the extracted higher-order rate constants (listed in Table 1) are, strictly speaking, moderated by the photon-to-free-charge-carrier conversion ratio φ (0 ≤ φ ≤ 1). However, given the low exciton binding energy of the FAPbI_3_ film compared to thermal energies (consistent with previous reports),^72^ we expect this ratio to be near unity at room temperature.

We find that the extracted higher-order recombination constants are clearly dependent on the FAPbI_3_ film processing route. Values for *k*_2_ (listed in Table 1) decrease significantly from that for the TA FAPbI_3_ control film (*φk*_2_ = 1.2 × 10^–10^ cm^3^ s^–1^) to those for films produced by the AAC routes (*φk*_2_ = 0.25 × 10^–10^ cm^3^ s^–1^ with DMF/DMSO, and *φk*_2_ = 0.24 × 10^–10^ cm^3^ s^–1^ with MASCN). This observed trend may be caused by two mechanisms. First, changes in crystallinity may affect *k*_2_ through altered photon outcoupling.^69^ However, the trend is perhaps too abrupt to make this the dominant mechanism (see a comparison of trends in crystallite size and *k*_2_ shown in Table 1). Second, the changes in *k*_2_ may be mostly affected by the extent to which quantum confinement is present. For the FAPbI_3_ control film, the clear presence of quantum-confined domains may enhance the electronic density of states near the band edges because of the decreased electronic dimensionality, thus increasing the level of bimolecular electron–hole recombination. For the FAPbI_3_ films produced via the AAC route, QC is strongly suppressed, leading to significantly lower values of *k*_2_. Similar trends are observed for Auger recombination constant *k*_3_ with the FAPbI_3_ control film exhibiting the highest value, while the magnitude is lower and comparable for both films produced via the AAC route. Given that Auger recombination has often been found to be enhanced in systems with lower electronic dimensionality (though the presence of impurities may also play a role), this finding again appears to corelate with the presence of electronic confinement.^73−75^ We note that these observations of increased higher-order recombination constants in systems of restricted dimensionality are similar to what has been observed previously in two-dimensional Ruddlesden–Popper lead iodide perovskites.^75^

We further use the OPTP photoconductivity data to extract values for charge-carrier mobilities, which are important predictors of the transport properties in device applications. Here, we leverage the direct relationship^76,77^ between photoconductivity and the measured value of the relative change in terahertz electric field transmission Δ*T*/*T* (see Figure S13). Immediately after photoexcitation, the photoconductivity is proportional to the product of the photogenerated density of electrons and holes (*n*_0_) and their respective mobilities. An electron–hole sum mobility value may therefore be extracted under the knowledge of *n*_0_, subject to photon-to-charge branching ratio φ (see section 13 of the Supporting Information and Figure S14 for full details). The resulting mobility values (φμ) are listed in Table 1 and illustrated graphically in Figure 4a for the three types of FAPbI_3_ films. The film fabricated through the MASCN route displays an excellent mobility of 49 cm^2^ V^–1^ s^–1^, roughly twice the value recorded for the FAPbI_3_ control film (25 cm^2^ V^–1^ s^–1^), with the DMF/DMSO route also offering an enhanced mobility of 39 cm^2^ V^–1^ s^–1^. These findings highlight the powerful effects of Lewis base-assisted crystallization on transport properties, which can be attributed to two critical factors. First, the AAC route results in films with a significantly lower density of grain boundaries (see SEM and XRD analysis) and defects (see values of *k*_1_), which decrease the extent of charge-carrier scattering and facilitate more efficient charge transport. Second, the FAPbI_3_ control film contains a significant amount of nanoconfined domains that may act as scattering centers or potential barriers impeding charge-carrier transport and substantially decreasing the overall mobility.^77,78^ Such nanoconfined domains are markedly reduced in the DMF/DMSO crystallized FAPbI_3_ and completely eliminated in the MASCN crystallized film, resulting in the significantly increased mobilities observed in films produced via the AAC route.

**Figure 4:**
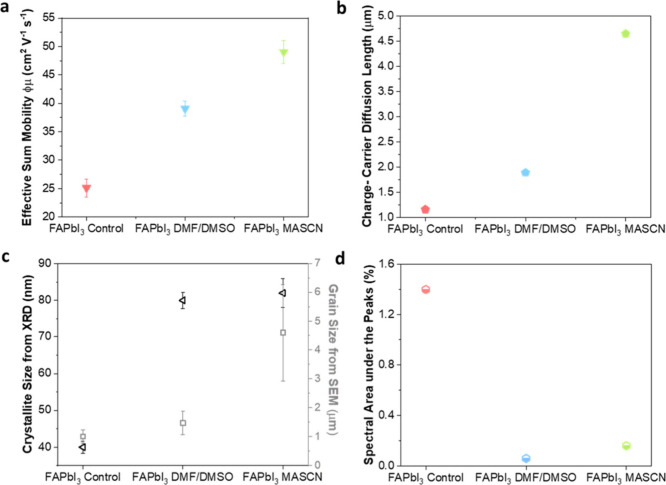
Correlation among charge-carrier transport metrics, film morphology, and quantum confinement effects for FAPbI_3_ films produced through three methods. (a) Effective electron–hole sum mobilities and (b) charge-carrier diffusion lengths for FAPbI_3_ films obtained from analysis of fluence-dependent OPTP transients, for a set charge-carrier density of 10^16^ cm^–3^. (c) Crystallite size (as obtained from XRD patterns) and upper limit of the grain size (as obtained from SEM measurements). (d) Parameter capturing the extent of quantum confinement: percentage of the area associated with the absorption peaks arising from quantum confinement relative to the total area under the absorption coefficient spectrum.

Using the charge-carrier recombination and transport parameters extracted from these measurements, we are able to determine the charge-carrier diffusion lengths (*L*_D_) for the FAPbI_3_ films produced through the three routes, using the following expression:^76^
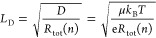
2where the term *R*_tot_ encompasses contributions from Auger, bimolecular, and SRH recombination and can be defined^70^ as *R*_tot_ = *n*^2^*k*_3_ + *nk*_2_ + *k*_1_ and *D* is the diffusion coefficient derived from the Einstein relation that links it to charge-carrier mobility as *D* = μ*k*_B_*T*/e.^76^ Plots depicting the prominence of *R*_tot_ and the interplay of the different recombination pathways at different values of *n* are provided in the Supporting Information (Figure S15). Because *R*_tot_ depends on the charge-carrier density, *L*_D_ will also, and we here determine the diffusion length for the approximate conditions prevalent under 1.5AM solar irradiation, assuming a charge-carrier density^79^ of 10^16^ cm^–3^. Figure 4b illustrates that the derived charge-carrier diffusion lengths for the FAPbI_3_ films progressively increase from the control film to the DMF/DMSO-crystallized film, ultimately reaching their maximum in the film produced via MASCN-assisted crystallization. These improvements are associated with both the reduction in the extent of charge-carrier recombination and enhancements in mobilities discussed above. Overall, we find a clear positive correlation between these trends in enhanced mobility and *L*_D_ values (Figure 4a,b) and those of increased crystallite/grain size (Figure 4c). These trends also correlate well with a reduced incidence of intrinsic quantum confinement (Figure 4d) calculated by taking the ratio of the integral area under the associated absorption peaks to the integral area under the absorption spectrum for a set energy range, as described previously.^46,47^ It should be further emphasized that while the FAPbI_3_ MASCN film shows a slightly increased area ratio compared to that of the DMF/DMSO sample, this holds no scientific relevance concerning the QC strength. As discussed in previous sections, the film exhibits no confinement effects, and the extracted features can be attributed to noise. The area ratio for FAPbI_3_ MASCN is presented solely for the purpose of a relative comparison.

Overall, we find that the aerosol-assisted crystallization method induces significantly improved optoelectronic and transport properties in FAPbI_3_ resulting from improved crystallinity and lower strain. Importantly, these improvements in the morphology and crystalline quality also have a direct impact on the presence of QC features, which are peculiar to FAPbI_3_. A recent statistical study, based on an extensive cross-literature analysis covering 244 articles and 825 photovoltaic devices incorporating FAPbI_3_ films, has shown that the presence of QC detrimentally correlates with device efficiency.^49^ Similarly, the AAC method had recently been shown^44^ to be associated with the improved photovoltaic device performance of FAPbI_3_ and is here demonstrated to result in suppressed QC effects. While the exact structural phases giving rise to such effects remain unidentified, they may pave the way for tailored material design on the nanoscale through the use of AAC. Here, elimination of QC effects may be the target for PV application; however, we note that such self-forming nanostructures could potentially prove to be useful for alternative applications such as light-emitting devices. Overall, these findings again highlight how lattice strain in these soft semiconductors may induce new phases that have distinct electronic properties.

In conclusion, this study demonstrates the effectiveness of the AAC method in fabricating high-quality FAPbI_3_ films with enhanced structural and optoelectronic properties. Compared with the standard thermal annealing approach, AAC-grown films exhibit larger grain sizes, reduced internal strain, and a substantial reduction in QC effects, which may hinder charge transport. A broad range of time-resolved spectroscopic analyses confirm that these improvements in crystalline quality directly translate to enhanced optoelectronic performance, with lower charge-carrier recombination rates, extended diffusion lengths, and enhanced mobility. The promising outcomes of this investigation suggest that the AAC method holds significant potential, offering a compelling solution to the enduring challenges in high-quality film fabrication for FAPbI_3_-based solar cells and paving the way for notable efficiency gains.

## Supporting Information Available

Sample preparation, experimental details, estimation of grain and crystallite size, Elliott fits, decoupling of peaks, correlation analysis between the peak positions across the films, fluence-dependent TRPL and OPTP analysis, and extraction of photoconductivity and mobility from OPTP transients (PDF)
