# Supplementary material for: Aerosol-Assisted Crystallization Lowers Intrinsic Quantum Confinement and Improves Optoelectronic Performance in FAPbI3 Films
Source: J Phys Chem Lett. 2025 Feb 21;16(9):2212–22. doi: 10.1021/acs.jpclett.5c00041 (PMC11891977; doi:10.1021/acs.jpclett.5c00041)
Supplement: Supplementary file 1 — jz5c00041_si_001.pdf [file jz5c00041_si_001.pdf]

Supporting Information

Aerosol-Assisted Crystallization Lowers Intrinsic Quantum Confinement and Improves Optoelectronic Performance in $FAPbI_3$ Films

Gurpreet Kaur,[1,†] Madsar Hameed,[2,3,†] Jae Eun Lee,[1] Karim A. Elmestekawy,[1] Michael B. Johnston,[1] Joe Briscoe,[2] and Laura M. Herz[1,4,]*

[1] Clarendon Laboratory, Department of Physics, University of Oxford, Oxford OX1 3PU, United Kingdom

[2] School of Engineering and Materials Science, Queen Mary University of London, Mile End Road, London E1 4NS, United Kingdom

[3] Department of Chemical & Polymer Engineering, University of Engineering & Technology Lahore, Faisalabad Campus, 3.5km, Khurrianwala – Makkuana By-Pass, Faisalabad, Pakistan

[4] Institute for Advanced Study, Technical University of Munich, D-85748, Garching, Germany

[†] These authors contributed equally

Contents

1. Sample fabrication 4

2. Experimental methods 5

Scanning electron microscopy 5

X-ray diffraction 5

Fourier transform infrared spectroscopy - absorption measurements 5

Steady-state photoluminescence (SSPL) measurements 6

Time correlated single photon counting (TCSPC) 6

Optical-pump terahertz-probe spectroscopy (OPTP) measurements 6

3. SEM grain size distribution 8

4. X-ray diffraction- crystallite size 9

5. Deducing absorption coefficient spectra from transmittance and reflectance 11

6. Elliott fitting 11

7. Extraction of the high energy peak features – baseline fitting 15

8. Quadratic behaviour of the decoupled peaks 16

9. Examination of the relationship between decoupled peak positions across the different $FAPbI_3$ films 17

10. Fluence-dependent time resolved photoluminescence (TRPL) transients 22

11. Fluence-dependent optical-pump terahertz-probe (OPTP) spectroscopy transients.. 23

12. Comparative analysis of TRPL data fitting with two different models.................. 27

Stretched exponential fitting model 27

Extraction of monomolecular recombination rates (k_1) by including k_2 obtained from OPTP decay curves 28

13. Deduction of photoconductivity and effective charge-carrier mobility from OPTP plots.. 32

14. Recombination rates as a function of charge-carrier density.................................. 37

1. Sample fabrication

The precursor solutions for $FAPbI_3$ films were prepared by dissolving equimolar concentrations (1.25 mol dm^{-3}) of PbI_2 (99.99% TCI) and FAI (Sigma Aldrich) in a mixed solvent of DMF and N-Methyl-2-pyrrolidone (NMP) in a volume ratio of 7:3. The solution was stirred at 50 °C until the precursors were dissolved. The solution was filtered through a 0.45 micron PTFE filter before deposition. Substrates of z-cut quartz, measuring 13 mm in diameter and 2 mm in thickness, were cleaned with water, acetone, and isopropanol. Then oxygen plasma treatment (5min, 90W) was used to further clean and activate the substrate surface and 0.40μL $FAPbI_3$ based perovskite precursor solution was dropped onto each substrate and spun at 3600rpm for 20s. At the 9th second, 0.5 ml diethyl ether was dripped onto the spinning substrate. The control films were then dried on a hot plate at 100°C for 2 min and then annealed at 150°C for 20 min in a nitrogen-filled glovebox.

For aerosol assisted crystallization (AAC), films were dried at 100°C for 2min in a nitrogen filled glovebox, then aerosol was generated through a piezoelectric ultrasonic mist generator and aerosol was introduced into the reactor using a nitrogen flow at 0.3LPM. The pre-dried films were treated for 2.5 min by using an aerosol generated from either DMF/DMSO (9:1) or a 1mg/mL solution of MASCN in DMF.

2. Experimental methods

Scanning electron microscopy

A thin gold layer of approximately 10 nm was applied to all films before imaging. The images were then captured using a FEI Inspect-F field emission scanning electron microscope, set to a working voltage of 3 kV.

X-ray diffraction

To confirm that the films crystallized in the desired cubic phase, XRD diffraction patterns were collected over a Bragg angle range of 10° to 45° using a Panalytical X'Pert powder diffractometer equipped with a Copper X-ray source (Cu – Kα, operated at 40 kV and 40 mA, λ = 1.5406 Å). Tilt corrections for all samples were made using the reference peak corresponding to the z-cut quartz substrate at 2θ = 16.431°. Before proceeding with analysis of the XRD data, background corrections were applied using HighScore Plus software to mitigate any potential inaccuracies in the measured values. The XRD pattern used as a reference in this study was simulated using VESTA software, utilizing the CIF file for cubic $FAPbI_3$ available at the following GitHub repository: [https://github.com/WMD-group/hybrid-perovskites/blob/fe4b188d5c7549050d9994c64bc86636968addd6/2014_cubic_halides_PBEsol/FAPbI3.cif]

Fourier transform infrared spectroscopy - absorption measurements

In order to collect the reflectance (R) and transmittance (T) spectra from the different samples, we have used a Fourier Transform Infrared Spectrometer (FTIR) from Bruker (Model: Vertex 80v). We have used two different source-detector configurations for

collection of data over the desired range of energies spanning from UV towards NIR. For collection in UV range, we have used a Xe lamp as the incident light source in conjunction with a gallium phosphide (GaP) detector (beam splitter: CaF_2), while for collection in visible domain, we have used a Xe source together with a silicon diode detector. All measurements have been done under vacuum (4 mbar).

Steady-state photoluminescence (SSPL) measurements

Steady-state PL emission from the films was recorded following excitation with a 398 nm diode laser from PicoQuant (Model – LDH-D-C-405M) with its setting in continuous wave mode (fluence 70 mW/cm^2). Subsequent to photoexcitation of the sample, the emitted PL was gathered and directed into a grating spectrometer (Model – SP-2558 from Princeton Instruments), dispersed, and then detected by a silicon based intensified charge-coupled device (ICCD, Model- PI-MAX4 from Princeton Instruments). All the measurements have been conducted in low pressure conditions, inside a vacuum cell ($\approx 10^{-2}$ mbar).

Time correlated single photon counting (TCSPC)

Time-resolved photoluminescence transients for the given samples were obtained using the same setup as for steady-state PL measurements, but with pulsed excitation at repetition rate of 1MHz, and detection by a photon counting detector (PDM series from Micro Photon Devices). The timing of the photon detection was controlled by a PicoHarp 330 event timer. The excitation photon energy was same as that for SSPL.

Optical-pump terahertz-probe spectroscopy (OPTP) measurements

Optical-Pump THz-Probe (OPTP) experiments were carried out using a Spectra Physics Mai Tai-Empower-Spitfire Pro Ti^{3+}: Sapphire regenerative amplifier, which produces pulses

with temporal width of 35fs and with their output wavelength centred at 800 nm with a 5 kHz repetition rate. The optical pump excitation was achieved by frequency doubling the laser output through a β-BBO (Barium Borate) crystal to generate 400 nm pulses. THz probe pulses were generated using a spintronic emitter consisting of 1.8 nm $Co_{40}Fe_{40}B_{20}$ layered between 2 nm of tungsten and 2 nm of platinum on a quartz substrate.[1] The experiments were done in transmission mode. THz pulse detection was carried out using the principle of free-space electro-optic sampling by having the gate pulse (a small portion of the output from the amplifier) incident on a 1 mm thick (110)- ZnTe crystal overlapping with the THz pulse. The resulting change in the gate pulse's polarization, directly related to the strength of the THz field, is detected using a quarter-wave plate, Wollaston prism, and a pair of balanced photodiodes. The timing between the pump and THz pulses is adjusted using optical delay stages. The FWHM spatial extent for the pump and THz beams at the sample spot were measured to be 3.5 mm and 0.9 mm, respectively. The measurements were done under vacuum conditions ($<10^{-1}$ mbar).

3. SEM grain size distribution

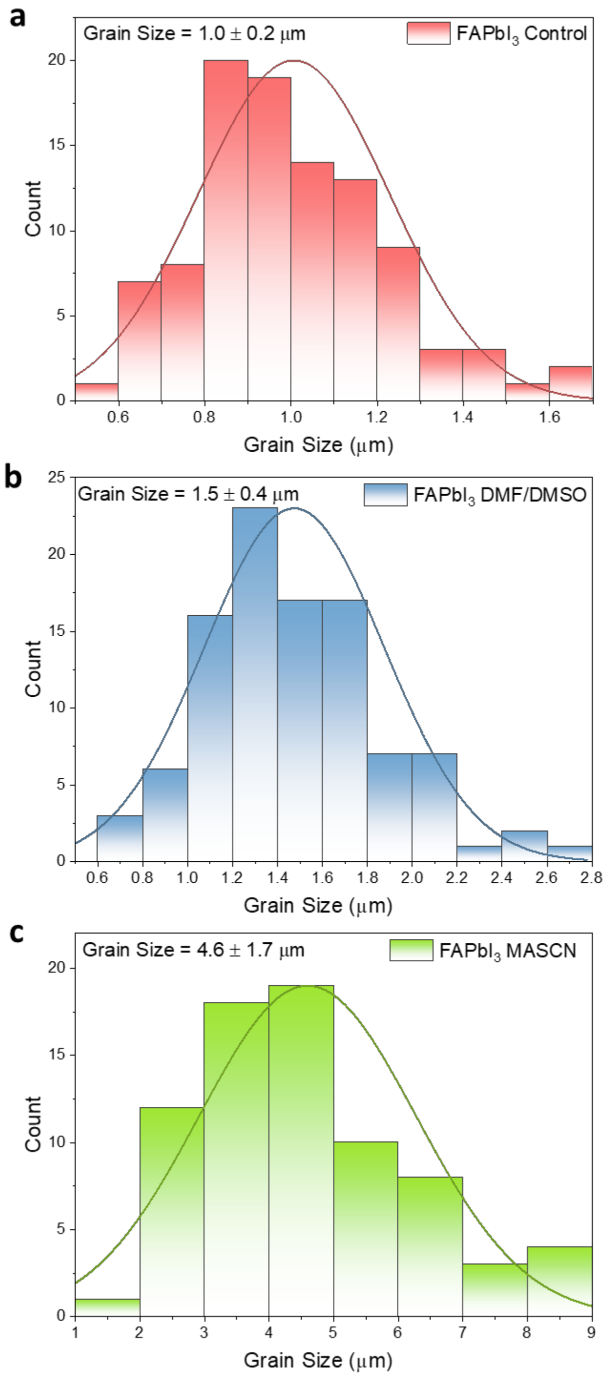

Figure S1. Grain size distribution extracted (using ImageJ software) from the SEM images provided in the main text.

4. X-ray diffraction- crystallite size

To estimate the crystallite size from the XRD patterns, we employed the standard Scherrer equation, using the full-width at half maximum (FWHM) of the diffraction peaks. This method provides an assessment of the crystallite size based on the assumption that the peak broadening is primarily due to finite crystallite size rather than strain or defects. For the analysis, we chose well-defined diffraction peaks corresponding to major crystal planes, specifically the (100) and (200) reflections. The peaks were fitted using pseudo-Voigt functions to ensure accurate determination of the FWHM. The broadening of these peaks was corrected by subtracting the instrumental broadening (obtained from the silicon reference) from the observed FWHM values. To assess the instrument broadening, we followed the same method as previously described,[2,3] using the diffraction pattern from a reference silicon sample (Figure S2a). FWHM values for the Si reference were plotted as a function of 2θ, and a quadratic equation in the form $y=ax^2+c$ was fitted to the data. This allowed us to estimate the instrument broadening (β_{inst}) at different scattering angles. The resulting plot and fit for the Si reference are presented in Figure S2b.

Figure S3 displays the FWHM plots for the chosen diffraction peaks for all the $FAPbI_3$ films after the instrumental corrections.

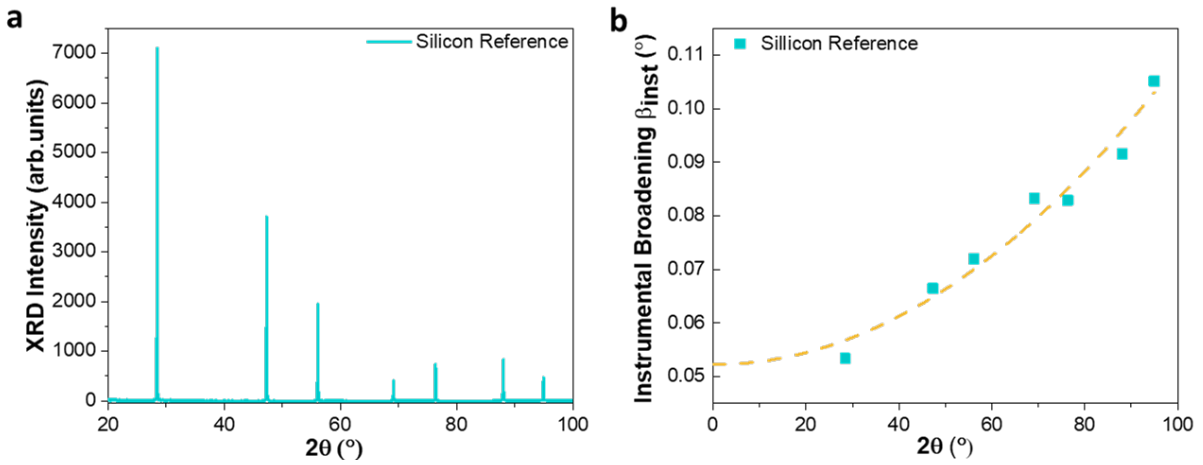

Figure S2. (a) XRD pattern for Silicon (reference) measured to determine the instrumental broadening. (b) Instrument Broadening curve for the X-ray diffractometer deployed in the current study for data acquisition. The solid dark cyan squares mark the FWHM of the peaks observed for the Si reference in (a). The orange curve represents a quadratic fit ($ax^2 + c$) to the data points.

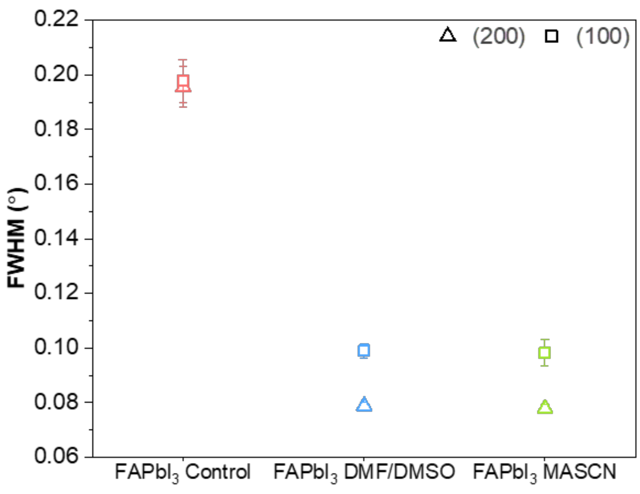

Figure S3. FWHM of the (100) and (200) peaks observed in X-ray diffraction patterns for the different $FAPbI_3$ films. The values have been extracted by Pseudo Voigt fitting of the peaks and after removing the instrumental broadening effects.

The corrected broadening was then applied to the Scherrer equation:

$$D = \frac{K\lambda}{\beta cos\theta} \quad \text{Eq. S1}$$

where D represents the average crystallite size, K is the Scherrer constant (typically 0.9 for spherical particles), λ is the X-ray wavelength (1.5406 Å for Cu Kα radiation), β is the corrected FWHM in radians, and θ is the Bragg angle corresponding to the peak. The crystallite size calculations were made from the (100) reflection.

5. Deducing absorption coefficient spectra from transmittance and reflectance

Using the measured Reflectances (R) and Transmittances (T), we extracted the absorption coefficient (α) spectra deploying the following relation:[4]

$$\alpha = \frac{-1}{d} log_{10}\left(\frac{T}{1-R}\right) \quad \text{Eq. S2}$$

Here d stands for the thickness of the sample deposited over the quartz substrate, $T = T_{sample}/T_{reference}$ and $R = R_{sample}/R_{reference}$. We have used a blank (uncoated) quartz substrate as the reference for transmission ($T_{reference}$) (since we have used a quartz substrate) and a silver mirror as reference for reflectance when measuring in visible–near-infrared region and an aluminium mirror for the UV domain. For all the films studied here, their thickness 'd' is measured to be approximately 400 nm. The measurements of film thicknesses have been made deploying a Veeco Dektak 150 surface profiler.

6. Elliott fitting

The absorption onset in the absorption coefficient spectrum was modelled using Elliott's theory, which provides a detailed framework to describe the optical absorption of semiconductors near their band edge.[5–8] Elliott's model accounts for both excitonic effects

and continuum states from unbound electron-hole pairs, giving a comprehensive picture of the absorption behaviour.

The energy-dependent absorption coefficient α(E) is expressed as the sum of two contributions:

$$\alpha(\mathrm{E}) = \alpha_{\mathrm{X}}(\mathrm{E}) + \alpha_{\mathrm{C}}(\mathrm{E}) \qquad \text{Eq. S3}$$

Bound exciton contribution - $\alpha_X(E)$

The excitonic part of the absorption - $\alpha_X(E)$ arises from bound exciton states and is described by:

$$\alpha_X = \frac{b_0}{E}\sum_{n=1}^{\infty}\frac{4\pi E_B^{3/2}}{n^3}\delta(E - (E_g - \frac{E_B}{n^2})) \qquad \text{Eq. S4}$$

- b_0 is a proportionality constant that includes the transition matrix element between the conduction and valence bands.
- The sum represents the weighted contribution from excitonic states with quantum number *n*, and the energies are given by $E_g - \frac{E_B}{n^2}$, where E_g is the bandgap energy and E_B is the exciton binding energy.

Continuum state contribution - $\alpha_C(E)$

The absorption from the continuum, where the electron-hole pairs are not bound, is represented as:

$$\alpha_C(E) = \frac{b_0}{E}\left[\frac{(\frac{2\pi\sqrt{E_B}}{\sqrt{E-E_g}})}{1-exp(\frac{-2\pi\sqrt{E_B}}{\sqrt{E-E_g}})}\right]\frac{JDoS(E)}{c_o} \qquad \text{Eq. S5}$$

where:

- $JDoS(E) = c_0\sqrt{E - E_g}$ is the joint density of states for energies above E_g which accounts for the continuum states.
- The Coulomb enhancement factor increases the absorption of the continuum due to the Coulomb interaction between the unbound electrons and holes.

The joint density of states constant c_0 is given by:

$$c_0 = \left(\frac{2\mu}{\hbar^2}\right)^{\frac{3}{2}} \frac{2}{(2\pi)^2} \quad \text{Eq. S6}$$

where μ is the reduced effective mass of the electron-hole system, and $\hbar$ is the reduced Planck constant. This expression captures the continuum absorption above the bandgap for a direct-gap semiconductor.

<u>Incorporating homogeneous broadening (γ)</u>

The combined excitonic and continuum contributions are then convolved with a normal distribution to account for homogeneous broadening caused by electron-phonon interactions:

$$g(E) = \frac{1}{\sqrt{2\pi\gamma^2}} exp\left(-\frac{E^2}{2\gamma^2}\right) \quad \text{Eq. S7}$$

Where γ is the broadening parameter related to electron-phonon coupling. This convolution ensures that the broadening of the absorption edge is taken into account.

The resultant Elliott fits, along with the original data are presented in Figure S4, while the fitting parameters obtained from these fits are summarised in Table S1.

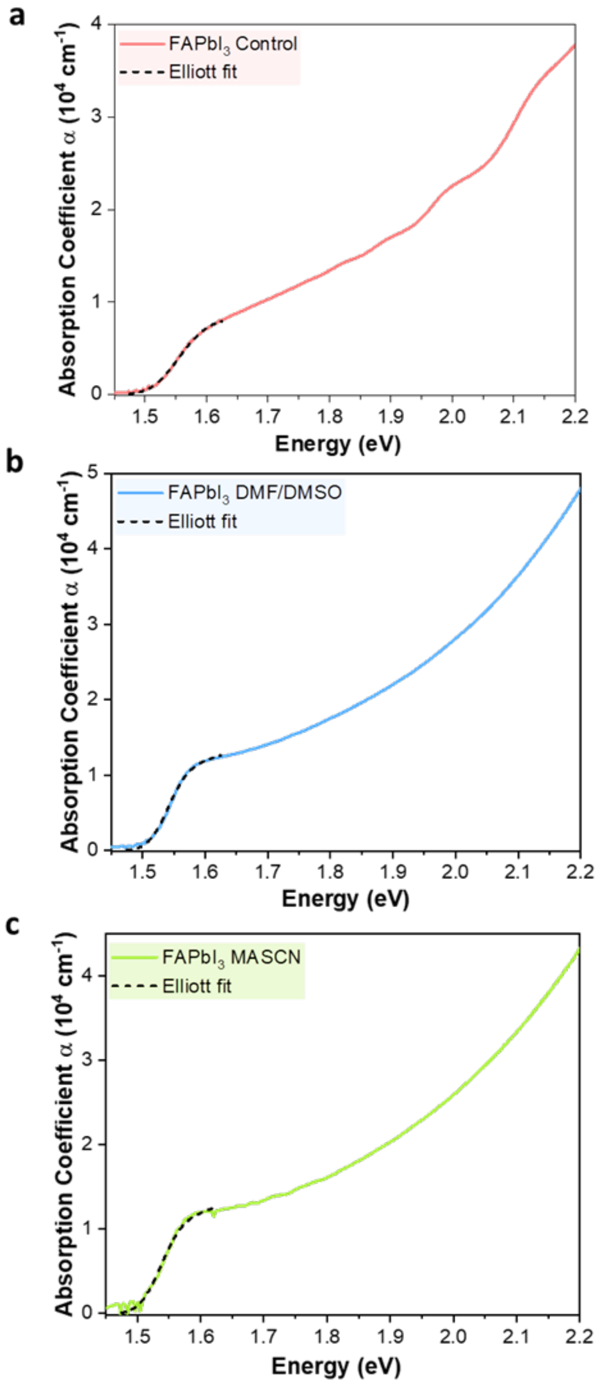

Figure S4. Elliott fits to the absorbance onsets used to extract the optical band gap energy (E_g), broadening parameter (γ) and exciton binding energy (E_B) tabulated in table S1.

Table S1. Parameters extracted as a result of Elliott fitting to the absorption onsets for $FAPbI_3$ films under investigation.

System	E_g (eV)	E_B (meV)	A (arb. units)	γ (meV)
$FAPbI_3$ Control	1.563	5	15.2	32.4
$FAPbI_3$ DMF/DMSO	1.552	5	23.5	25.8
$FAPbI_3$ MASCN	1.547	5	23.3	27.0

For fitting the band edge absorption data, exciton binding energy (E_B) has been globally fitted across all the samples (the obtained value is consistent with those reported in the literature) [9] while the other parameters – optical band gap (E_g), broadening(γ) and the spectrum amplitude (A) have been set as free parameters.

7. Extraction of the high energy peak features – baseline fitting

To enable a quantitative analysis of the superimposed peak features, it is necessary to separate them from the predominantly monotonous above-bandgap absorption. We have followed the same methodology as reported by Wright et al.[10] We began by applying a smoothing technique through local regression using weighted linear least squares with a second-degree polynomial. This produced a smooth curve (depicted in solid black line, Figure S5) that captured the overall trend in the data. Subtracting this curve from the experimental measurements allowed us to extract a distinct undulating component, where the minima aligned with the dips in the original data, effectively removing the influence of the steadily increasing absorption. A piecewise cubic spline was then used to interpolate between these minima, creating a baseline fit (shown in dashed deep red fit, Figure S5). By

subtracting this spline baseline from the original data, we successfully isolated the peaks, which provided a clearer basis for quantitative analysis. These peaks are examined in more detail in the subsequent sections of this study.

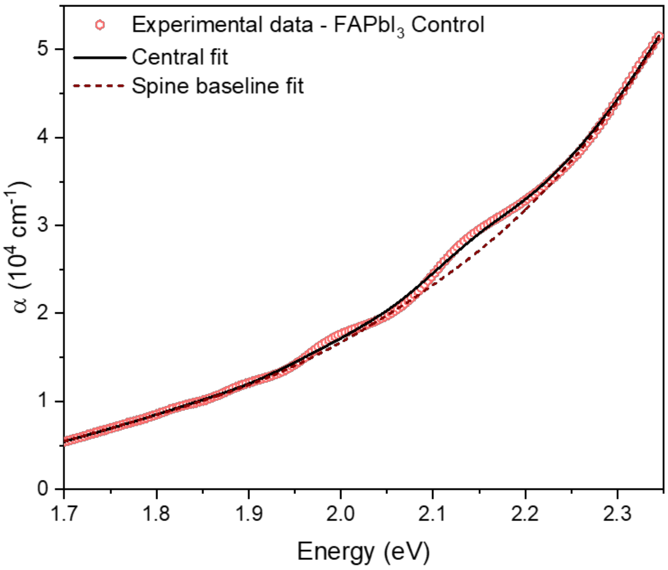

Figure S5. Absorption coefficient spectrum above the energy band gap, fitted with a phenomenological smoothing function that traces the centres of the peak features. This central fit is then used to generate a spline baseline that follows the troughs of the features.

8. Quadratic behaviour of the decoupled peaks

Figure S6 displays the absorption peak features associated with quantum confinement, that have been decoupled from the original absorption spectra. The excellent fit demonstrates conformity to the quadratic energy spacing for electronic confinement in an infinite potential well or superlattice,[10] suggesting the presence of intrinsic quantum confinement effects in the $FAPbI_3$ film prepared through the typical thermal annealing approach. The peak indexing is aligned with that used in previous reports for consistency.[10] In this case,

the indexing begins at 8, as additional low-energy peaks become apparent only at cryogenic temperatures, but are not observable at room temperature here.

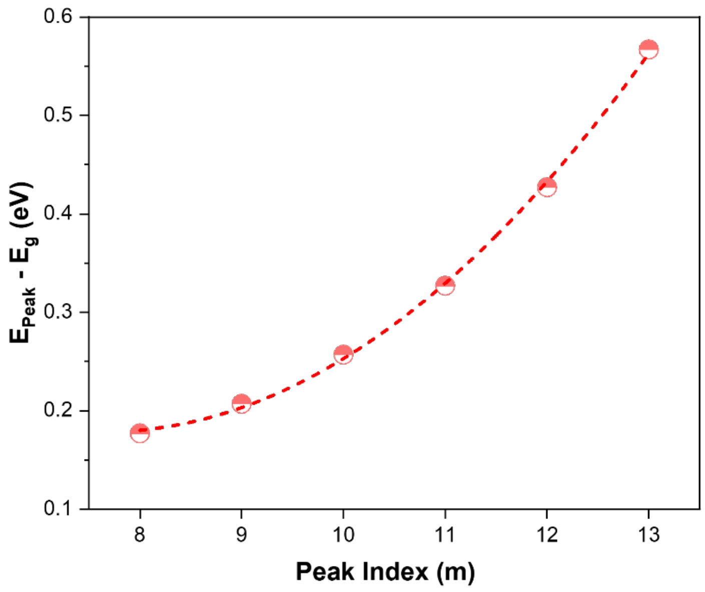

Figure S6. Variation of confinement energy, defined as the difference between the energy of the peaks (E_{Peak}) observed in the absorbance spectrum and optical band gap (E_g) plotted as a function of peak index for the thermally annealed $FAPbI_3$ Control film. The dashed line represents a quadratic fit to these data points.

9. Examination of the relationship between decoupled peak positions across the different $FAPbI_3$ films

Regarding the relative amplitudes of the decoupled peaks for the different $FAPbI_3$ films, the different y-axis scales clearly demonstrate that, even if the AAC-prepared films exhibit any undulations obscured by noise, they are significantly weaker than those observed in the $FAPbI_3$ Control film. When the extracted features are shown above each other on the

same spectra axis, they indicate a potential correlation in the peak positions across the different samples.

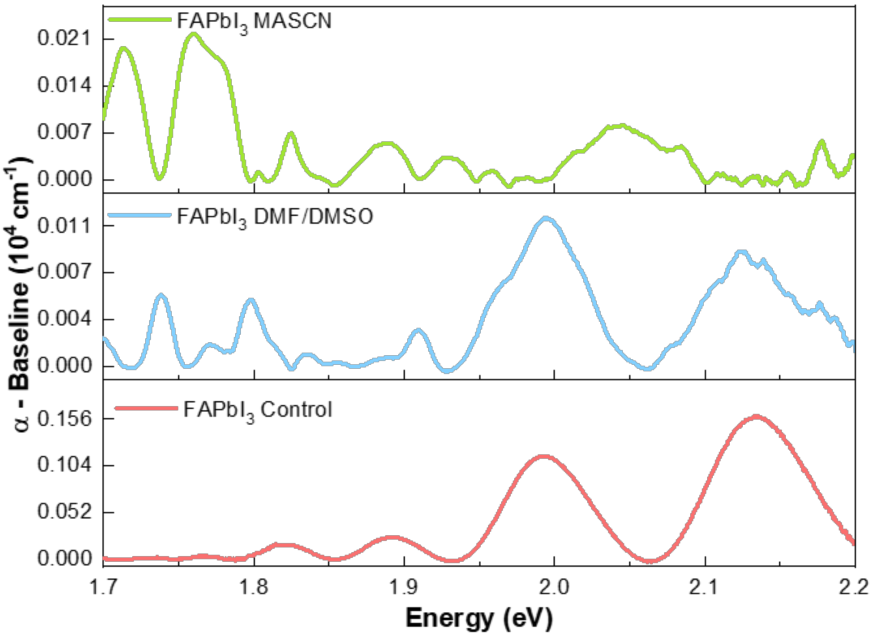

Figure S7. Peaks isolated from the absorbance spectra collected for $FAPbI_3$ films plotted separately in different panels covering the same energy axis and zoomed in for better visualization.

Correlation analysis for the peak positions of the extracted peaks

Figure S7 clearly demonstrates that among the three different $FAPbI_3$ systems, only the $FAPbI_3$ Control film exhibits clearly distinct and prominent peak features. In contrast, the undulations observed in the films prepared via the AAC route appear relatively insignificant. To determine whether similar features are present in the data for the AAC samples but perhaps buried under noise due to weakened quantum confinement effects (resulting in low amplitude and hence poor signal-to-noise ratio), we conducted a correlation analysis of the modulations obtained from all samples. By using a Pearson

correlation analysis,[11] we quantitatively assessed the similarity of spectral features across different samples. Consequently, we extracted Pearson correlation coefficients (ρ) among the undulations obtained for the different films, where the value of ρ between any two variables, say S1 and S2 can be calculated as:

$$\rho_{S1,S2} = \frac{\mathrm{cov}(S1,S2)}{\sigma_{S1}\sigma_{S2}} \qquad \text{Eq. S8}$$

where σ_{S1}, σ_{S2} stand for the standard deviation for the random variates S1, S2 and cov (S1, S2) is the covariance between S1 and S2. In the present case, the variates are the undulations or features extracted from the absorption data of different $FAPbI_3$ films prepared through different crystallization routes. From the correlation matrix which contains all these values of ρ (Figure S8), a moderate positive correlation is revealed between the observed features of the control film (just thermally annealed $FAPbI_3$) and that prepared by treatment of $FAPbI_3$ with DMF/DMSO vapours.

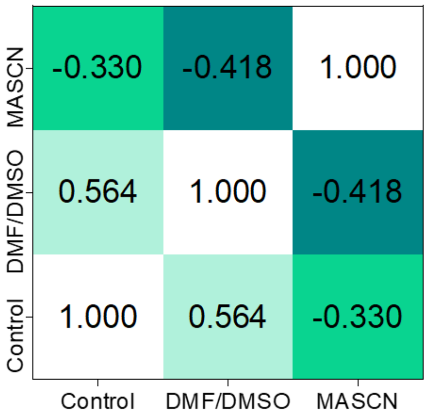

Figure S8. Correlation matrix describing the correlation between the energetic positions of the peaks dissociated from the absorbance spectrum. The entities contained in the matrix

are the Pearson correlation coefficients obtained upon conducting Pearson correlation analysis on the undulations/peaks in the absorption associated with quantum confinement.

Cross correlation analysis

Furthermore, the cross-correlation coefficients obtained reveal a commendable degree of positive correlation ($\rho \approx 0.8$) between decoupled peaks obtained for the $FAPbI_3$ Control film and the $FAPbI_3$ DMF/DMSO film if we account for a 0.011 eV spectral lag in the features (Figure S9). Notably, this shift matches the difference in energy band gap between the DMF/DMSO treated $FAPbI_3$ film and the just thermally annealed $FAPbI_3$ Control film.

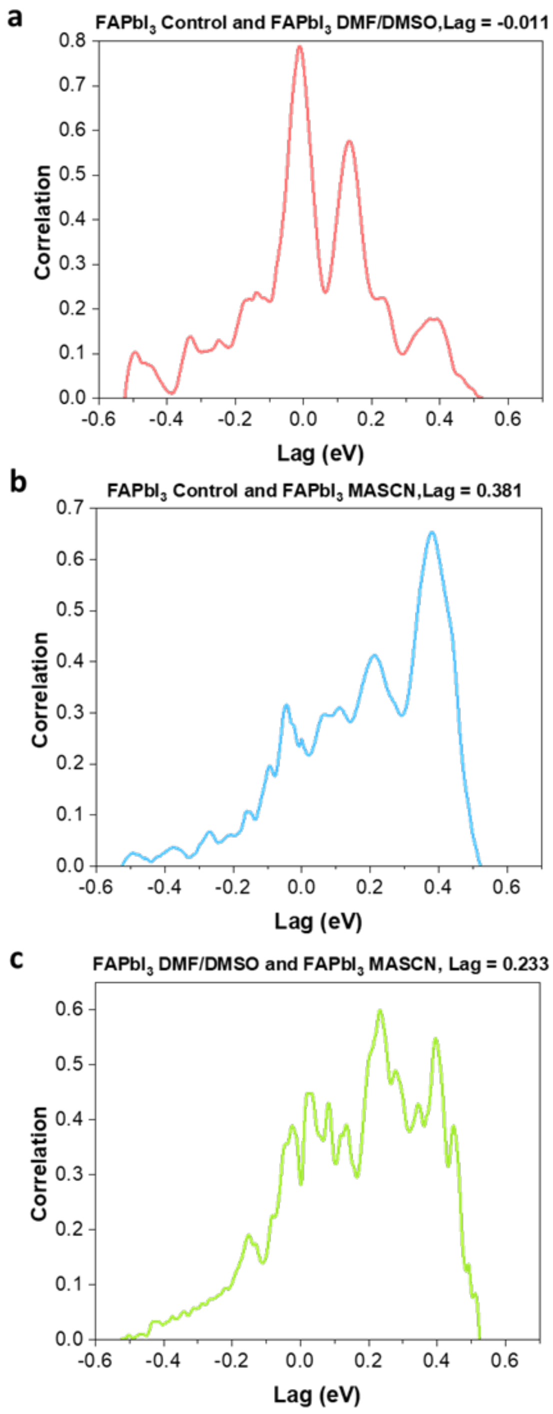

Figure S9. Results from cross correlation analysis conducted for each possible pair of $FAPbI_3$ films from the ensemble. "Lag" represents the amount in eV by which the decoupled peaks/total absorbance spectrum should be translated so that the peak positions

for the two systems being compared would hold good strong positive correlation (≈0.8-1) between themselves.

10. Fluence-dependent time resolved photoluminescence (TRPL) transients

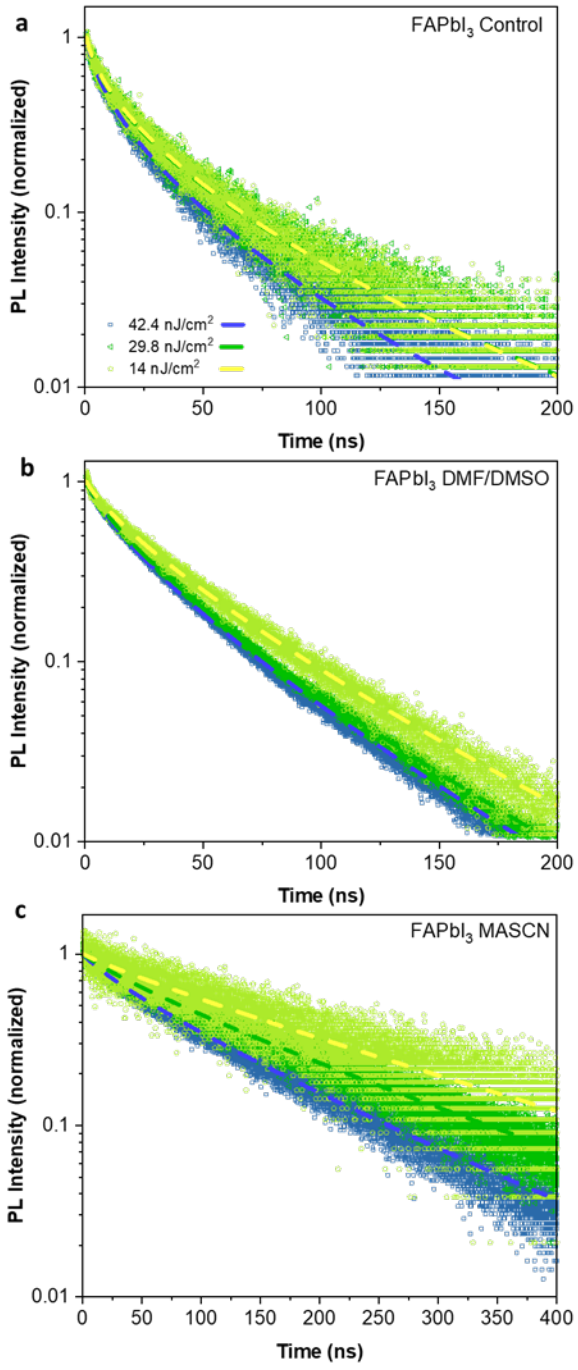

Figure S10. Fluence-dependent TRPL transients for (a) $FAPbI_3$ Control and for $FAPbI_3$ films crystallized through (b) DMF/DMSO-based and (c) MASCN-based AAC approach.

The legends in (a) also apply to (b) and (c). Open symbols are the experimental data set and the dashed lines are the result of stretched exponential fitting with the fit parameters given in Table S2.

Table S2. Parameters obtained by fitting the fluence dependent TRPL transients recorded for $FAPbI_3$ films with stretched exponential functions.

Sample	Fluence (nJ / cm^2)	β	τ (ns)	k_1 ($10^6 s^{-1}$)
$FAPbI_3$ Control	42.9	0.56	10.1 ± 0.2	59.5 ± 0.90
	29.8	0.56	13.0 ± 0.3	46.6 ± 1.10
	14	0.57	13.6 ± 0.3	45.6 ± 1.03
$FAPbI_3$ DMF/DMSO	42.9	0.74	23.3 ± 0.1	35.6 ± 0.12
	29.8	0.74	25.0 ± 0.1	33.2 ± 0.10
	14	0.77	31.3 ± 0.2	27.4 ± 0.16
$FAPbI_3$ MASCN	42.9	0.82	92.2 ± 0.5	9.7 ± 0.05
	29.8	0.84	126.0 ± 0.9	7.2 ± 0.11
	14	0.89	173.0 ± 2.4	5.5 ± 0.17

11. Fluence-dependent optical-pump terahertz-probe (OPTP) spectroscopy transients

Global fits to photoinduced transmission plots ($\Delta T/T$ plots) & extraction of apparent bimolecular and Auger recombination rates (φk_2 and $\varphi^2 k_3$)

As mentioned in the main text, the free charge-carrier recombination dynamics for any semiconductor system (if we ignore the diffusion and transport processes) can be described

by taking into account the first, second and third order decay mechanisms through the following rate equation:[12]

$$\frac{dn}{dt} = -k_3n^3 - k_2n^2 - k_1n \qquad \text{Eq. S9}$$

Here, $n(t)$ represents the free charge-carrier density and k_1, k_2, and k_3 are the rate constants for monomolecular, bimolecular band-to-band, and Auger recombination channels, respectively.

Further, the quantity 'n' is directly linkable to the experimentally determined parameter – optically induced change in THz field transmission $\Delta T/T(t) \equiv x(t)$ through the relation

$$n(t) = \varphi\, C\, x(t) \qquad \text{Eq. S10}$$

where C = $\tilde{n}_0/x(0)$ is the proportionality factor between initial THz response, x (0), and absorbed photon density $\tilde{n}_0$.This initial photon density $\tilde{n}_0$ can be calculated as per the following equation:

$$\tilde{n}_0 = \frac{E\lambda}{\mathrm{h}cA_{\mathrm{eff}}d}(1 - R_{\mathrm{PUMP}}(\lambda) - T_{\mathrm{PUMP}}(\lambda)) \qquad \text{Eq. S11}$$

where d is the thickness of the thin film, h is Planck's constant, c is the speed of light, R_{pump} (λ) is the reflectance and $T_{\mathrm{pump}}(\lambda)$ is the sample transmittance at the pump wavelength λ. The term E/A_{eff} represents the incident fluence, where E can be estimated from laser power incident on the sample for the given measurement and its repetition rate, and A_{eff} is the effective overlap area of the optical pump and probe beams. Furthermore, the effective overlap area A_{eff} can be calculated as:[13]

$$A_{eff} = \frac{\pi}{\ln(2)}(FWHM^2_{\text{pump}} + FWHM^2_{\text{probe}}) \qquad \text{Eq. S12}$$

Substituting eq. S10 into the rate equation eq. S9, we obtain the following relation:[14]

$$\frac{dn(t)}{dt} = -A_3x^3 - A_2x^2 - A_1x \qquad \text{Eq. S13}$$

with $A_1 = k_1$, $A_2 = C\varphi k_2$ and $A_3 = C^2\varphi^2k_3$. The numerical solutions to this ordinary differential equation (ODE) are applied globally to fit the decay profiles across all fluence levels, allowing for the extraction of the rate constants A_i. As the exact photon-to-free-charge conversion ratio φ cannot be determined from the present measurements, only the apparent recombination rates, φk_2 and $\varphi^2 k_3$ can be extracted. Since $0 \leq \varphi \leq 1$, the resulting values of k_2 and k_3 are necessarily underestimations of the true intrinsic bimolecular and Auger recombination rates. However, given that the exciton binding energy in $FAPbI_3$ is much smaller (5 meV, see above) than thermal energies at room temperature, excitons are likely to be fully dissociated and φ is approximately 1 in this case.

Given that the charge-carrier density is not uniform throughout the photoexcited film, we account for the exponential charge-carrier density profile induced by the pump beam (in accordance with the Beer Lambert's law for absorption). To model this, the sample is divided into 30 equally thick slices, and the decay dynamics are calculated for each slice individually, integrating over the entire film depth profile to provide a comprehensive picture of the recombination behaviour.

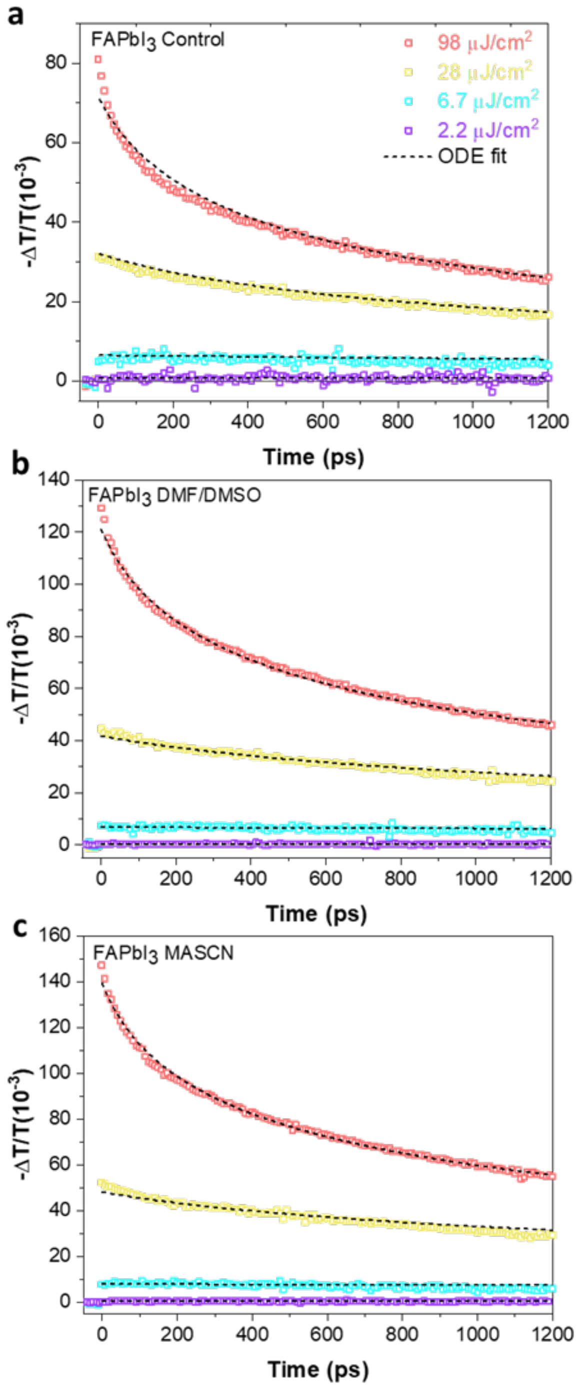

Figure S11. Fluence dependent OPTP dynamics for the thin $FAPbI_3$ films produced through different methods. Open symbols represent experimental data while the dashed

curves are fits to the typical equation that describes the time-dependent decay of charge-carrier density (explained in detail in the text) from where the bimolecular recombination rate constant k_2 and Auger recombination rate constant k_3 are extracted.

12. Comparative analysis of TRPL data fitting with two different models

Mono-exponential fitting does not accurately capture the essence of the PL decay for the present set of data. There could be two possible explanations for this: inhomogeneous distribution of the defect states leading to existence of local distribution of mono-exponential decay rates and contributions stemming from the bimolecular recombination persisting even at such low excitation fluences. To address this, we applied two independent models and made a comparative analysis: stretched exponential fitting as described in the main text (fluence dependent data shown in Figure S10 above) and a model that includes fitting the data to the analytic solution of the rate equation (including contribution from bimolecular recombination using the values extracted from fluence dependent OPTP fits but ignoring higher-order processes).

Stretched exponential fitting model

The stretched mono-exponential fitting appears to be nominally effective for the TRPL dataset here (Figure S10). However, a closer examination of the fitting results for the fluence-dependent TRPL data (Table S2) reveals a slight fluence dependence in the extracted time constants and corresponding k_1 values, suggesting a marginal, albeit not hugely significant, involvement of the higher-order processes.

Extraction of monomolecular recombination rates (k_1) by including k_2 obtained from OPTP decay curves

To further address this, we also accounted for any possible presence of bimolecular recombination processes by globally fixing k_2 as obtained from the OPTP analysis (fitting results provided in Table 1 in main text) and then extracted k_1. Given that Auger recombination (k_3) is insignificant under the incident fluences deployed in TRPL experiments (because k_3 extracted here from OPTP is of the order of 10^{-30}-10^{-31} cm^6/s), we set $k_3 = 0$ when considering the PL dynamics recorded under much lower fluences, which reduces Eq. S9 to :

$$\frac{dn}{dt} = -k_2 n^2 - k_1 n \qquad \text{Eq. S14}$$

<u>Analytical solution:</u>

To solve this equation, we perform variable separation:

$$\int \frac{dn}{n\left(1+\frac{k_2}{k_1}n\right)} = \int -k_1\, dt \qquad \text{Eq. S15}$$

This integration results in:

$$ln\left(\frac{n}{1+\frac{k_2 n}{k_1}}\right) = -k_1 t + A \qquad \text{Eq. S16}$$

Exponentiating both sides give,

$$\frac{n}{1+\frac{k_2 n}{k_1}} = Be^{-k_1 t} \qquad \text{Eq. S17}$$

Where $B = e^A$ is a constant. Solving for n(t) yields

$$n(t) = \frac{B}{e^{k_1 t - \frac{k_2 B}{k_1}}} \quad \text{Eq. S18}$$

Subsequently, substituting for $\alpha = \frac{B}{k_1}$, the equation becomes

$$n(t) = \frac{k_1 \alpha}{e^{k_1 t - k_2 \alpha}} \quad \text{Eq. S19}$$

To relate this to the initial charge-carrier density n_0, we use the condition at t=0, giving:

$$\frac{1}{\alpha} = \frac{k_1}{n_0} + k_2 \quad \text{Eq. S20}$$

This ensures that the solution recovers the correct initial condition, n(t=0) = n_0.

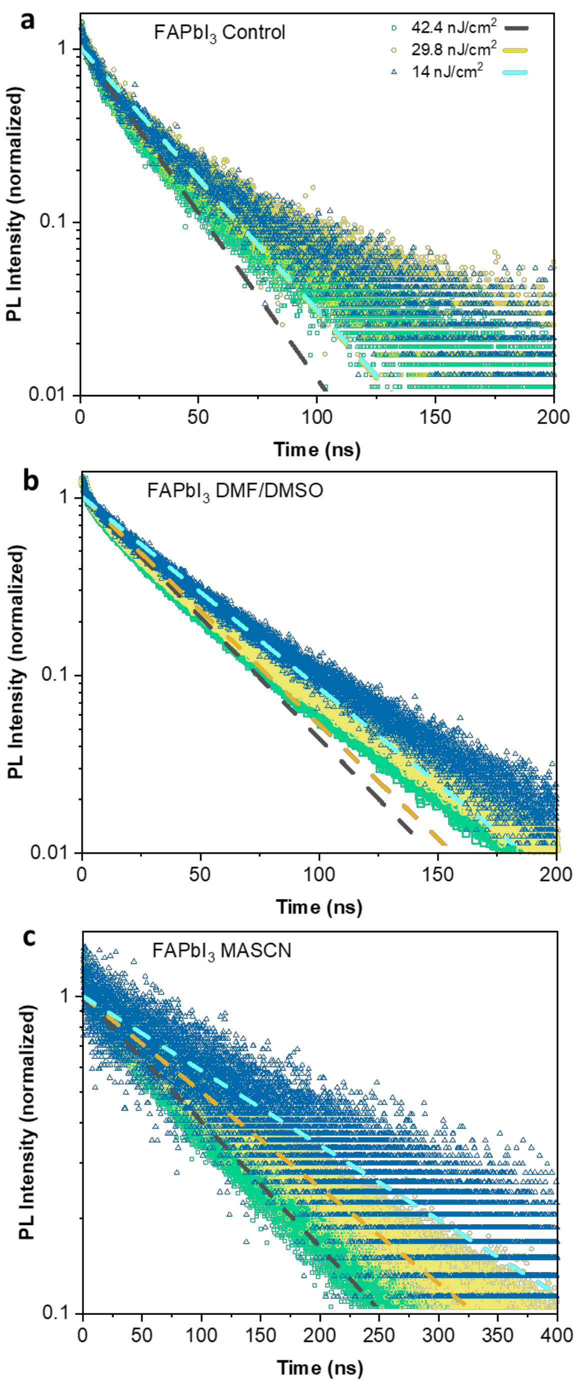

Figure S12. Fluence-dependent TRPL transients for (a) $FAPbI_3$ Control (b) $FAPbI_3$ DMF/DMSO and (c) $FAPbI_3$ MASCN films. The open symbols represent experimental data, while the dashed lines show the results of fitting the data to the analytical solution of the standard ordinary differential equation, which describes the decay of charge-carrier

density over time after photoexcitation (as explained in the text) (here k_2 was fixed to the value extracted from OPTP fits and setting $k_3 = 0$).

In this way, we obtain a simple analytic solution (Eq. S17) and use it to model the TRPL data. Here, k_1 was extracted for each fluence by fitting the transients, while globally fixing k_2 obtained from OPTP analysis for each sample. The fluence-dependent TRPL data along with the corresponding fits using this model here is presented in Figure S12 and the resulting recombination rate constants are given in Table S3.

Examining the extracted k_1 values, we observe that while the overall trend remains the same and therefore the inferences drawn, most values are smaller than those obtained (particularly at relatively higher fluences) when not considering any bimolecular recombination contribution. This reduction in the k_1 when including bimolecular recombination arises because the total recombination rate is distributed between both processes:

$$R_{total} = R_{mono} + R_{bi} = k_1 + k_2 n \qquad \text{Eq. S21}$$

When bimolecular recombination is excluded from the analysis, all recombination is attributed to the monomolecular rate, leading to an overestimated k_1. However, when bimolecular recombination is included, the total recombination is more accurately described by both the k_1 and the k_2 n term. As a result, part of the recombination previously assigned to the monomolecular process is reassigned to the bimolecular process, leading to a lower k_1 value.

Table S3. SRH recombination rate constants obtained from TRPL traces fitting by fixing k_2 and setting k_3 as zero.

Sample	Fluence (nJ / cm^2)	k_1 (10^6 s^{-1})
$FAPbI_3$ Control	42.9	44.1 ± 0.17
	29.8	35.1 ± 0.16
	14	34.9 ± 0.15
$FAPbI_3$ DMF/DMSO	42.9	31.4 ± 0.07
	29.8	29.6 ± 0.06
	14	24.8 ± 0.05
$FAPbI_3$ MASCN	42.9	9.1 ± 0.01
	29.8	6.9 ± 0.01
	14	5.4 ± 0.02

13. Deduction of photoconductivity and effective charge-carrier mobility from OPTP plots

The change in the transmission of the THz field ($\Delta T/T$) (essentially the differential change in THz transmission in the presence of the incident optical pump and that with the pump OFF) can be linearly related to the sheet photoconductivity (ΔS) of the sample through the following relation[15]

$$\Delta S = \frac{-\varepsilon_0 c(\eta_A + \eta_B)\Delta T}{T} \quad \text{Eq. S22}$$

where c stands for the speed of light in vacuum, ε_0 implies the free space permittivity, η_A and η_B denote the refractive index of the materials on either side of the deposited perovskite layer (here, we use $\eta_A = 1$ (air) and $\eta_B = 2.13$(quartz)). Please note that this relationship is valid only when the film thickness is significantly less than the smallest wavelength contained in the THz pulse (according to the thin film approximation) [13] a condition that is met in the present case.

Furthermore, the effective charge-carrier mobility ($\varphi\mu$) is in turn directly related to photoconductivity as[12]

$$\varphi\mu = \frac{\Delta S\, A_{\mathrm{eff}}}{N\mathrm{e}} \qquad \text{Eq. S23}$$

where A_{eff} stands for the effective area of overlap between the incident pulses, i.e. the optical pump and THz probe given by Gaussian spatial distribution of their intensities, e is the elementary charge and N is the number of charge carriers generated in the film following photoexcitation.

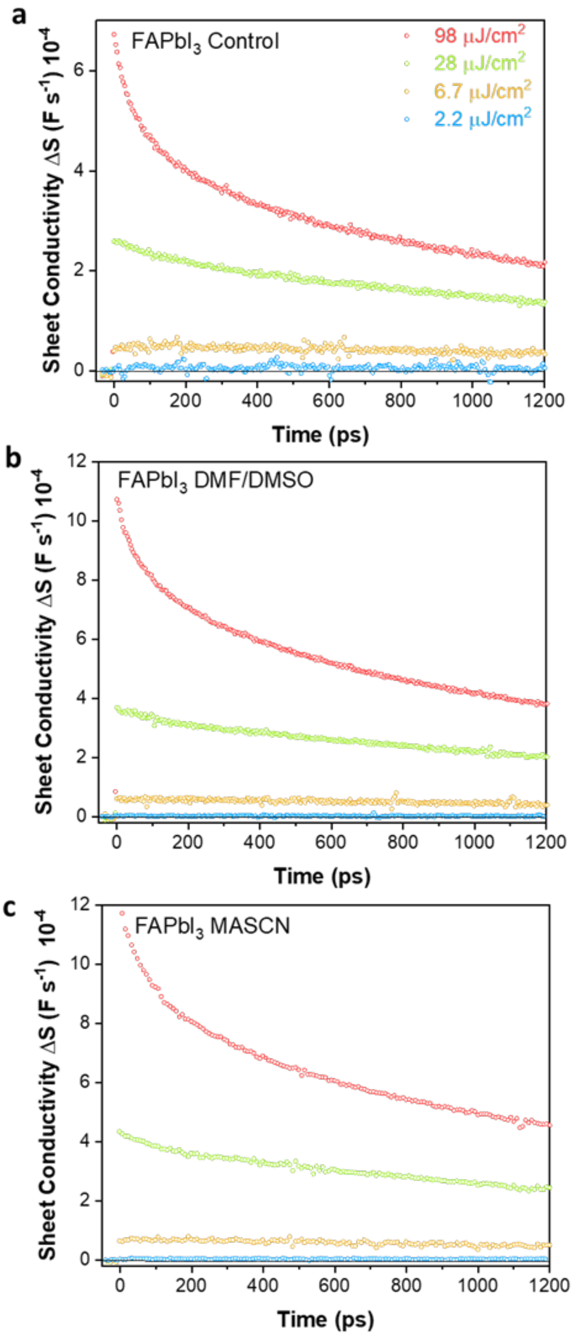

Figure S13. Photoconductivity decay transients monitored as a function of different fluences for $FAPbI_3$ produced through the three methods. The values of sheet photoconductivity were determined using the experimentally observed quantity $-\Delta T/T$ using Eq. S22.

Thus, as suggested by Eq. S23, in order to derive the effective electron-hole sum mobilities one needs to know the number of photo-excited charge carriers 'N'. This number in turn can be determined deploying using the following equation[15]

$$N = \varphi \frac{E\lambda}{\mathrm{hc}} (1 - R_{\mathrm{Pump}} - T_{\mathrm{Pump}}) \qquad \text{Eq. S24}$$

Here E implies energy per pulse for the incident optical pump for wavelength λ (here λ= 400 nm), R_{Pump} implies sample reflectivity and T_{Pump} refers to the transmittance recorded from FTIR measurement at this specific λ.

We have assumed φ to be equal to unity, implying a complete conversion of incident photons to free electrons and holes, since the exciton binding energy of $FAPbI_3$ film is very low compared to thermal energies. Furthermore, the values obtained here reflect contributions from both holes and electrons, making it impossible to isolate their individual contributions. Consequently, the effective mobility values we have here represent the combined mobilities of electrons and holes.

Thus, upon substituting the expressions from Eq. S22, S23 into Eq. S24 we obtain a relationship that directly links the effectivity charge-carrier mobility with the experimentally measured quantity $\Delta T/T$,

$$\varphi\mu = \frac{-\varepsilon_0 c(\eta_A + \eta_B)}{Ee\lambda(1 - R_{\mathrm{Pump}} - T_{\mathrm{Pump}})} A_{\mathrm{eff}} \mathrm{h}c\left(\frac{\Delta T}{T}\right) \qquad \text{Eq. S25}$$

From this equation, we then determine mobility values across the different samples (Figure S14). For calculating the charge-carrier mobilities, we determine the initial absolute value of the OPTP amplitude immediately after excitation ($\Delta T/T$) before the charge-carrier

density has declined or redistributed as a result of charge-carrier recombination, photon reabsorption and diffusion. We included data from all measured fluences because the proportional relationship between $\Delta T/T$ and fluence was observed to hold consistently across all fluence ranges.

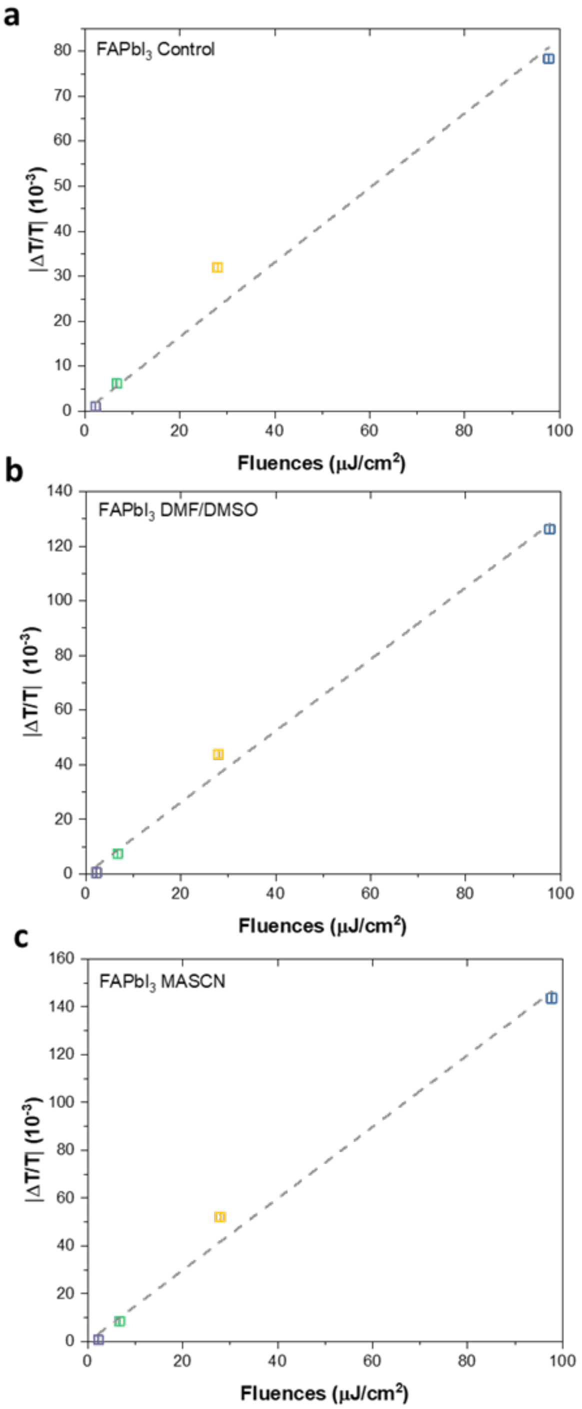

Figure S14. Peak $|\Delta T/T|$ values (open squares) recorded immediately following photoexcitation, plotted as a function of the incident fluence for each $FAPbI_3$ thin film. The dashed lines are linear fits to these data points following the constraint that these fits should necessarily pass through the origin.

14. Recombination rates as a function of charge-carrier density

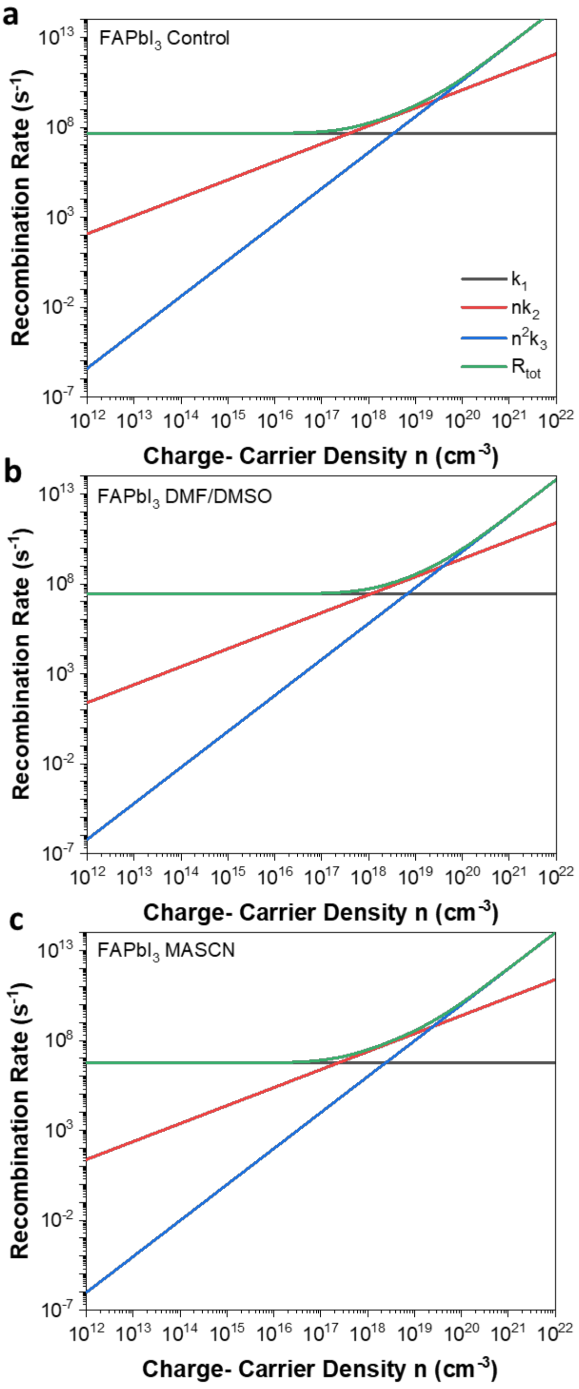

Figure S15. Total recombination rate (R_{tot}) as a function of charge-carrier density (n) for the studied $FAPbI_3$ films, with individual recombination pathways represented by their respective terms. The plots illustrate the contribution and interplay of these pathways across the examined n range. The legend in (a) also applies to (b) and (c).

As evident from the plots, at low n (< 10^{16} cm^{-3}), monomolecular recombination dominates chiefly. However, as n increases, higher-order recombination terms start to become dominant – firstly bimolecular recombination gradually takes over, with the contribution from k_1 declining. Once n reaches close to 10^{20} cm^{-3}, Auger recombination becomes the dominant mechanism and the contribution stemming from monomolecular recombination becomes insignificant.

References

(1) Wagner, F. M.; Melnikas, S.; Cramer, J.; Damry, D. A.; Xia, C. Q.; Peng, K.; Jakob, G.; Kläui, M.; Kičas, S.; Johnston, M. B. Optimised Spintronic Emitters of Terahertz Radiation for Time-Domain Spectroscopy. *J Infrared Millim Terahertz Waves* **2023**, *44* (1–2), 52–65. https://doi.org/10.1007/s10762-022-00897-9.

(2) Lohmann, K. B.; Patel, J. B.; Rothmann, M. U.; Xia, C. Q.; Oliver, R. D. J.; Herz, L. M.; Snaith, H. J.; Johnston, M. B. Control over Crystal Size in Vapor Deposited Metal-Halide Perovskite Films. *ACS Energy Lett* **2020**, *5* (3), 710–717. https://doi.org/10.1021/acsenergylett.0c00183.

(3) Hargreaves, J. S. J. Some Considerations Related to the Use of the Scherrer Equation in Powder X-Ray Diffraction as Applied to Heterogeneous Catalysts. *Catalysis, Structure and Reactivity* **2016**, *2* (1–4), 33–37. https://doi.org/10.1080/2055074X.2016.1252548.

(4) Elmestekawy, K. A.; Gallant, B. M.; Wright, A. D.; Holzhey, P.; Noel, N. K.; Johnston, M. B.; Snaith, H. J.; Herz, L. M. Photovoltaic Performance of $FAPbI_3$ Perovskite Is Hampered by Intrinsic Quantum Confinement. *ACS Energy Lett* **2023**, *8* (6), 2543–2551. https://doi.org/10.1021/acsenergylett.3c00656.

(5) Elliott, R. J. Intensity of Optical Absorption by Excitons. *Physical Review* **1957**, *108* (6), 1384–1388. https://doi.org/10.1103/PhysRev.108.1384.

(6) Yang, Y.; Yang, M.; Li, Z.; Crisp, R.; Zhu, K.; Beard, M. C. Comparison of Recombination Dynamics in $CH_3NH_3PbBr_3$ and $CH_3NH_3PbI_3$ Perovskite Films: Influence of Exciton Binding Energy. *Journal of Physical Chemistry Letters* **2015**, *6* (23), 4688–4692. https://doi.org/10.1021/acs.jpclett.5b02290.

(7) Kentsch, R.; Scholz, M.; Horn, J.; Schlettwein, D.; Oum, K.; Lenzer, T. Exciton Dynamics and Electron-Phonon Coupling Affect the Photovoltaic Performance of the $Cs_2AgBiBr_6$ Double Perovskite. *Journal of Physical Chemistry C* **2018**, *122* (45), 25940–25947. https://doi.org/10.1021/acs.jpcc.8b09911.

(8) Buizza, L. R. V.; Wright, A. D.; Longo, G.; Sansom, H. C.; Xia, C. Q.; Rosseinsky, M. J.; Johnston, M. B.; Snaith, H. J.; Herz, L. M. Charge-Carrier Mobility and Localization in Semiconducting Cu_2AgBiI_6 for Photovoltaic Applications. *ACS Energy Lett* **2021**, *6* (5), 1729–1739. https://doi.org/10.1021/acsenergylett.1c00458.

(9) Galkowski, K.; Mitioglu, A.; Miyata, A.; Plochocka, P.; Portugall, O.; Eperon, G. E.; Wang, J. T. W.; Stergiopoulos, T.; Stranks, S. D.; Snaith, H. J.; Nicholas, R. J. Determination of the Exciton Binding Energy and Effective Masses for Methylammonium and Formamidinium Lead Tri-Halide Perovskite Semiconductors. *Energy Environ Sci* **2016**, *9* (3), 962–970. https://doi.org/10.1039/c5ee03435c.

(10) Wright, A. D.; Volonakis, G.; Borchert, J.; Davies, C. L.; Giustino, F.; Johnston, M. B.; Herz, L. M. Intrinsic Quantum Confinement in Formamidinium Lead Triiodide Perovskite. *Nat Mater* **2020**, *19* (11), 1201–1206. https://doi.org/10.1038/s41563-020-0774-9.

(11) Lee Rodgers, J.; Nicewander, W. A. Thirteen Ways to Look at the Correlation Coefficient. *Am Stat* **1988**, *42* (1), 59–66. https://doi.org/10.1080/00031305.1988.10475524.

(12) Buizza, L. R. V.; Crothers, T. W.; Wang, Z.; Patel, J. B.; Milot, R. L.; Snaith, H. J.; Johnston, M. B.; Herz, L. M. Charge-Carrier Dynamics, Mobilities, and Diffusion Lengths of 2D–3D Hybrid Butylammonium–Cesium–Formamidinium Lead Halide Perovskites. *Adv Funct Mater* **2019**, *29* (35), 1902656. https://doi.org/10.1002/adfm.201902656.

(13) Xia, C. Q.; Peng, J.; Poncé, S.; Patel, J. B.; Wright, A. D.; Crothers, T. W.; Uller Rothmann, M.; Borchert, J.; Milot, R. L.; Kraus, H.; Lin, Q.; Giustino, F.; Herz, L. M.; Johnston, M. B. Limits to Electrical Mobility in Lead-Halide Perovskite Semiconductors. *Journal of Physical Chemistry Letters* **2021**, *12* (14), 3607–3617. https://doi.org/10.1021/acs.jpclett.1c00619.

(14) Rehman, W.; McMeekin, D. P.; Patel, J. B.; Milot, R. L.; Johnston, M. B.; Snaith, H. J.; Herz, L. M. Photovoltaic Mixed-Cation Lead Mixed-Halide Perovskites: Links between Crystallinity, Photo-Stability and Electronic Properties. *Energy Environ Sci* **2017**, *10* (1), 361–369. https://doi.org/10.1039/c6ee03014a.

(15) Righetto, M.; Wang, Y.; Elmestekawy, K. A.; Xia, C. Q.; Johnston, M. B.; Konstantatos, G.; Herz, L. M. Cation-Disorder Engineering Promotes Efficient

Charge-Carrier Transport in $AgBiS_2$ Nanocrystal Films. *Advanced Materials* **2023**, *35* (48), 2305009. https://doi.org/10.1002/adma.202305009.
